# Two-dimensional silk

**DOI:** 10.1126/sciadv.ado4142

**Published:** 2024-09-18

**Authors:** Chenyang Shi, Marlo Zorman, Xiao Zhao, Miquel B. Salmeron, Jim Pfaendtner, Xiang Yang Liu, Shuai Zhang, James J. De Yoreo

**Affiliations:** ^1^ Physical Sciences Division, Pacific Northwest National Laboratory, Richland, WA 99352, USA.; ^2^ Department of Materials Science and Engineering, University of Washington, Seattle, WA 98195, USA.; ^3^ Molecular Engineering & Sciences Institute, University of Washington, Seattle, WA 98195, USA.; ^4^ Materials Sciences Division, Lawrence Berkeley National Laboratory, Berkeley, CA 94720, USA.; ^5^ Department of Materials Science and Engineering, Stanford University, Stanford, CA 94305, USA.; ^6^ Department of Materials Science and Engineering, University of California, Berkeley, CA 94720, USA.; ^7^ Department of Chemical and Biomolecular Engineering, North Carolina State University, Raleigh, NC 27695, USA.; ^8^ College of Ocean and Earth Sciences, Xiamen University, Xiamen 361005, China.

## Abstract

Despite the promise of silk-based devices, the inherent disorder of native silk limits performance. Here, we report highly ordered two-dimensional silk fibroin (SF) films grown epitaxially on van der Waals (vdW) substrates. Using atomic force microscopy, nano–Fourier transform infrared spectroscopy, and molecular dynamics, we show that the films consist of lamellae of SF molecules that exhibit the same secondary structure as the nanocrystallites of native silk. Increasing the SF concentration results in multilayers that grow either by direct assembly of SF molecules into the lamellae or, at high concentrations, along a two-step pathway beginning with a disordered monolayer that then crystallizes. Scanning Kelvin probe measurements show that these films substantially alter the surface potential; thus, they provide a platform for silk-based electronics on vdW solids.

## Teaser

Two-dimensional crystalline layers of silk self-assemble on van der Waals substrates.

## INTRODUCTION

Silk is a natural protein-based material that has been used by humankind for over 5000 years (*1*). One of the two main components, silk fibroin (SF) has been exploited in recent decades for its ability to self-assemble into a range of fibril-based architectures that exhibit exceptional mechanical, optical, and excellent biocompatibility and biodegradability (*2*–*4*). Potential bioelectronic applications have been explored in which SF films are interfaced with van der Waals (vdW) solids, metals, or oxides, offering impressive electronic performance for next-generation thin-film transistors, memristors, human-machine interfaces, and sensors (*4*–*7*), provided that strategies can be developed to address challenges associated with the mismatch between the soft SF proteins and the hard, planar substrates (*6*).

Previous research to understand the formation and function of SF films has identified the molecular architecture achieved via self-assembly as an essential factor in their properties (*4*, *5*, *8*, *9*). In general, synthesis is carried out through self-assembly in solution, followed by deposition on a substrate. As with most self-assembling protein systems, SF assembly in solution relies on inter- and intramolecular interactions associated with backbone-backbone hydrogen bonds and amino acid side-chain chemistry (*10*). However, the introduction of a surface with which proteins have some affinity has been shown to give rise to new two-dimensional (2D) phases that do not exist in the bulk solution due to the introduction of protein-surface interactions, including electrostatic interactions due to the inherent charge of many surfaces, hydrophobic interactions when the surfaces are nonpolar, modified protein-solvent interactions due to solvent structuring at the interface, and chemical bonds including metal coordination complexes, π-π interactions, and hydrogen bonds (*11*–*16*). Despite the fact that these interactions have been shown to create unique, highly ordered 2D phases, this phenomenon remains unexplored in the case of SF films.

**Fig. 1.**
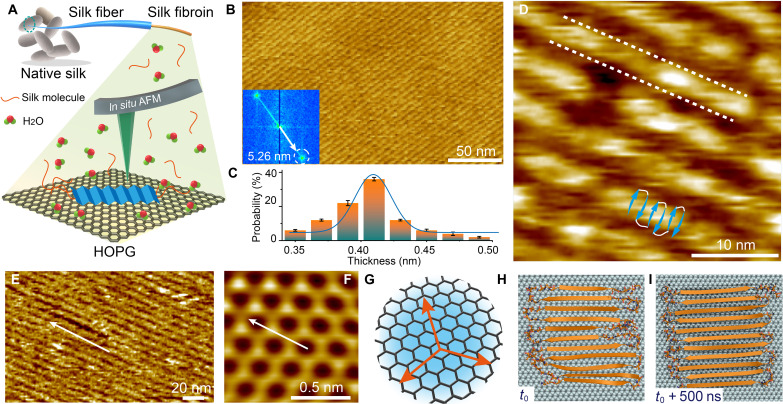
The structure of 2D SF lamellae grown epitaxially on HOPG. (**A**) Scheme of SF assembly on HOPG characterized by in situ AFM. (**B**) AFM image of a lamellar SF monolayer on HOPG formed at an SF concentration of 0.05 μg/ml and an incubation time of 30 min. Inset shows corresponding fast Fourier transform (FFT) pattern. (**C**) Thickness distribution of the lamellae. (**D**) AFM image of SF lamellae showing distinct segments. The illustration consisting of solid blue arrows and white connectors indicates how the antiparallel array of individual SF β strands from the MD simulations maps onto the lamellae and is not intended to be dimensionally accurate. The blue arrows correspond the orange arrows in (H). Dashed white lines delineate the boundary of single lamellae. The SF concentration is 0.05 μg/ml, and the incubation time is 60 min. (**E** and **F**) AFM images of (E) SF lamellar structure and (F) underlying HOPG lattice showing that lamellae lie along the armchair lattice direction as illustrated in (**G**). The SF concentration is 0.05 μg/ml, and the incubation time is 30 min. (**H** and **I**) Simulations of SF β sheet binding to HOPG. An SF protein with β sheet conformation initially aligned along the armchair direction (H) remains structured in that alignment after 500 ns (I). To determine the geometric relationship between the SF lamellae and the underlying HOPG lattice structure (F), the SF film (E) was scratched off using contact mode AFM in liquid.

Here, we report the discovery of a new 2D crystalline phase of SF self-assembled on vdW solids with an epitaxial relationship to the underlying lattice. This 2D phase forms when the SF concentration in solution is above a minimum value needed to drive nucleation and below the concentration at which the SF begins to assemble in the solution. Using in situ atomic force microscopy (AFM) (Fig. 1A), synchrotron-based nano–Fourier transform infrared (IR) spectroscopy (FTIR), and molecular dynamics (MD), we find that this structure is formed through surface-directed assembly and folding of the SF molecules, with a final architecture composed of fully ordered monolayers of β sheet lamellae, distinct from the disordered 3D network of β sheet nanocrystallites that defines silk fibril formed in bulk. Moreover, we show that assembly proceeds along two distinct pathways: direct epitaxial growth at low SF concentrations and a two-step process passing through a transient disordered phase at high concentration. These findings provide a mechanistic understanding of assembly for this canonical biomaterial that can enable efficient approaches to designing and fabricating highly oriented silk-based bioelectronics.

## RESULT

### Crystalline silk monolayers through heteroepitaxy

Aqueous SF solutions were made by the dissolution in deionized water of lyophilized SF powder, prepared via the well-established LiBr extraction method (*17*). In situ circular dichroism spectroscopy demonstrates that the SF primary structure in solution immediately after preparation is unstructured with a high content of random coils and negligible β sheet conformation (fig. S1) (*18*). Unlike the 3D fibrils formed when SF solutions are allowed to age, when incubated with highly oriented pyrolytic graphite (HOPG), these SF solutions form well-ordered lamellae (Fig. 1B) with a periodicity of about 5 nm (Fig. 1B and fig. S2) and a height of 0.42 nm (Fig. 1C and fig. S3), which is comparable to the thickness of a single SF β sheet secondary structure in fibril (*19*). AFM imaging also shows that the individual rows are composed of distinct segments (Fig. 1D) and lie along the three armchair lattice directions of HOPG (Fig. 1, E to G, and fig. S4). This orientational preference was also predicted by MD simulations in which the SF β sheet bounding to HOPG was found to be energetically favorable along these directions (Fig. 1I and figs. S5 and S6) (*20*), and secondary structure was stable over 500 ns. Images collected shortly after introduction of SF solution show that even the smallest observable nuclei are already ordered into the lamellar structure and lie along the preferred lattice directions from their first appearance (fig. S7). These observations demonstrate that the SF exhibits 2D lattice–matched, epitaxial growth on HOPG.

Measurements of the longitudinal growth rate 𝑣 of the SF lamellae (i.e., parallel to the lamellae) in this low-concentration regime show that it is proportional to the SF concentration *C* (fig. S8). Thus, longitudinal growth follows the classic behavior expected when growth is limited by attachment kinetics—that is *v* = ωβ(*C* − *C*_e_), where *C*_e_ = 0.007 μg/ml is the equilibrium concentration (i.e., the concentration at which *v* = 0), ω is the molecular volume of an SF unit in the lamella, and β is the kinetic coefficient (*21*). Because these lamellar films form row by row, as shown previously for lamellar peptide films on MoS_2_, there is no free energy barrier to nucleation, and, thus, lamellar islands begin to nucleate as soon as the concentration exceeds *C*_e_ (*11*). Note that the value of β largely reflects the activation barrier to attachment and the value of *C*_e_ largely reflects the difference between the barriers to attachment and detachment. Without knowledge of the attempt frequencies for either process, the values cannot be determined from data at a single temperature.

The lateral growth rate is substantially lower—6× at *C* = 0.025 μg/ml—reflecting the need to nucleate a new row adjacent to the existing rows (fig. S9). This difference in growth rates is likely due to the folded conformation of β sheets within the lamellae combined with stronger binding to the ends of the lamellae than to the sides. The structural ordering in the parallel β strands of an existing lamella should provide a low-energy site for subsequent attachment and folding of a new SF molecule, essentially acting like a template for the folding of the attaching SF molecule, as has been shown to be the case for other proteins that transition from disordered monomeric states to folded states during crystallization (*15*, *22*), thus reducing the activation barrier to attachment and, hence, to longitudinal growth. In contrast, to grow laterally, new rows must nucleate adjacent to existing ones to which the interaction is weak, leading to a larger energy barrier and slower growth. Similar discrepancies in growth rate have been observed for other systems of both organic molecules and peptides exhibiting highly anisotropic orthogonal interactions (*11*, *23*).

### Silk multilayers through homoepitaxy

**Fig. 2.**
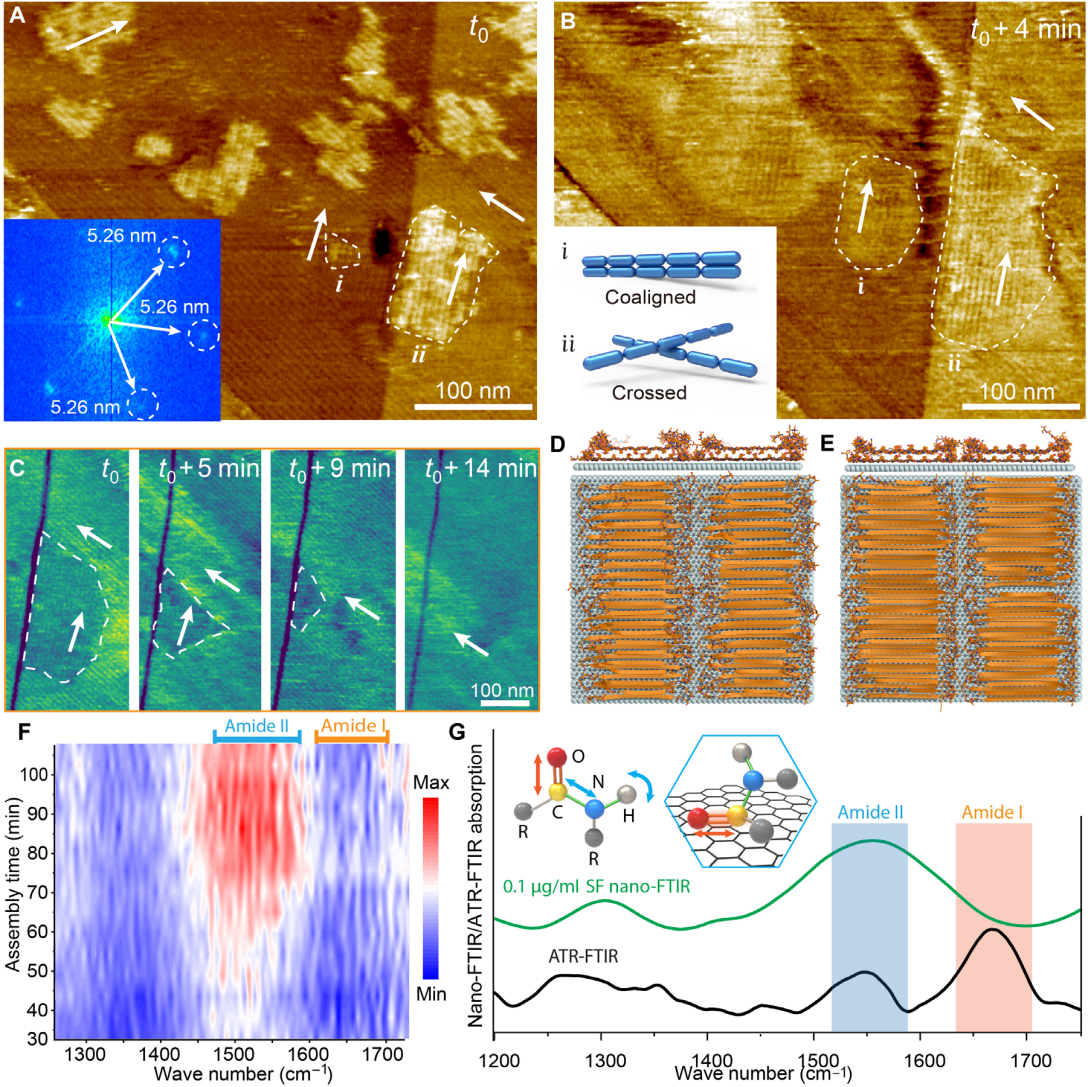
The structure of SF multilayers. (**A** and **B**) Two consecutive AFM images of multilayer SF lamellae. The SF concentration is 0.1 μg/ml, and the incubation time *t*_0_ is about 70 min. The inset in (A) shows the corresponding FFT pattern. White arrows indicate the three directions of the lamellae. The white dashed boundaries delineate two domains (i and ii) of lamellae overlaying the lower layer. The inset in (B) illustrates the two possible stacking configurations, coaligned and at 120° angles. (**C**) In situ AFM phase images of lamevvvllae, showing the replacement of a crossed domain by a coaligned domain. The white arrows indicate the orientation within and outside of the crossed domain, which is delineated by the white dashed boundaries. The SF concentration is 0.1 μg/ml, and the incubation time *t*_0_ is about 90 min. (**D** and **E**) MD simulations of bilayer SF lamellae with (D) face-to-face and (E) front-to-back packing. (**F**) Color map showing the time evolution of the in situ nano-FTIR spectra during SF assembly on graphene from an aqueous SF solution of 0.1 μg/ml. (**G**) In situ nano-FTIR spectra of SF lamellae and ex situ attenuated total reflection (ATR)–FTIR spectrum of dehydrated SF bulk material (see the section “In situ nano-FTIR characterization” in Materials and Methods for details). The insets illustrate the amide I and II vibration modes during IR absorption (left) and the expected vibration mode of the ordered amide group on graphene (right), both from the MD simulations of the SF configuration within the lamellae and the lack of an amide I signal in the nano-FTIR spectra.

With increased SF concentration (≥0.1 μg/ml), multiple lamellar layers formed after 50 to 60 min of incubation, with the upper layers again aligned along the armchair directions of the HOPG lattice (Fig. 2A, regions i and ii). These multilayers exhibited two types of stacking (Fig. 2, A and B). In the first, lamellae of one layer are both coaligned with (Fig. 2B, region i) and in precise registry with (fig. S10A) the lamellae of the underlying layer. As with the first monolayer, these coaligned lamellae have a height of ~0.42 nm (fig. S10C). In the second form of stacking, the upper and lower lamellae are crossed at an angle of 120°, although the height of the lamellae remains the same within error (inset in Fig. 2B).

In conventional silk crystallization from bulk solution, β sheet strands pack layer by layer to form nanocrystallites of coaligned strands that are interconnected within a disordered 3D network (*24*, *25*). Hence, the coaligned multilayers mimic the bulk crystallization pathway of the nanocrystallites. Moreover, within a given layer, the crossed lamellae are unstable relative to the coaligned lamellae and, thus, are eventually replaced by coaligned lamellae through the retreat of the former and the advance of the latter (Fig. 2C and fig. S11). This observation demonstrates that SF monomers are continually undergoing attachment and detachment at the ends of the lamellae and implies that there is some degree of misalignment between the amino acid residues of the upper and lower crossed lamellae that interfere with the hydrogen-bond network or the hydrophobic interactions between SF molecules. The resulting film comprises a highly organized sequence of lamellar layers in which β strands are coaligned within each layer and exhibit precise out-of-plane registry throughout the sequence.

To understand the molecular interactions between the lamellae of adjacent layers, we simulated two possible cases, one in which the β sheets stack face to face—i.e., the well-accepted “polar packing” of bulk silk (*26*)—and one in which they stack front to back (Fig. 2, D and E). The MD simulations predict that both configurations are stable; however, MD simulations of a bilayer with polar packing predict that the height of the second layer is 0.4 nm, similar to what is observed experimentally, while a bilayer with the β sheets stacked front to back is predicted to be more tightly packed and exhibit a height for the second layer of only 0.32 nm (table S1). Protein-protein Lennard-Jones interactions were similar. However, protein-solvent interactions were more favorable for the polar-packed configuration, suggesting that the stability is conferred by interactions with the solvent (fig. S12). Because these simulations were initialized in a packed configuration, it is not possible to conclude whether both cases are capable of forming. However, the corresponding heights imply that the observed lamellar films are consistent with the same polar packing believed to define the structure of bulk silk materials.

To understand the conformational evolution of the SF molecules during formation of the lamellae, we applied synchrotron-based in situ nano-FTIR to follow the change in secondary structure during assembly on graphene with nanometer resolution (see figs. S13 and S14 for schematic of the nano-FTIR setup and example of resulting data). The results show that, at an SF concentration of 0.1 μg/ml, the intensity of the peak in amide II region (1480 to 1600 cm^−1^) is first detectable by about 30 min, starts to increase markedly after 50 min of incubation, and reaches its maximum after 70 min. These timescales are consistent with AFM data collected at the same concentration (fig. S15). In contrast, only a weak signal from the water bending mode appears in amide I region (1600 to 1700 cm^−1^) (*27*, *28*), where attenuated total reflection (ATR)–FTIR data collected on a bulk silk sample records a strong peak (Fig. 2G). This effect is exclusively due to the polarization of the near-field IR beam, which is exclusively responsive to vibrational modes characterized by transition dipole moments perpendicular to the surface (*27*, *29*). The low ratio of the amide I band intensity to that of the amide II band indicates a preferential vibration of the C═O bond parallel to the surface (Fig. 2G), which is consistent with the C═O bond direction seen in the MD simulations (fig. S16) and implies a uniform configuration of the SF molecules within the lamellae. Together, these two features in the nano-IR spectra imply that the silk molecules themselves are well ordered within the lamellae.

The evolution in peak intensity of the amide bands over time reflects the structural rearrangement of the SF molecules during lamella formation. The initial increase in the amide II region indicates rapid development of the β sheet conformation. Conversely, the amide I region displays no changes in IR signal intensity, indicating that there are no unfolded proteins on the surface, even in the early stages of lamella formation. These observations imply that the SF proteins add to the lamellae directly from solution and adopt the β sheet structure at the time of attachment, leading to longitudinal growth (fig. S17), thus, providing compelling evidence that the assembly of the lamellae follows the conventional formation pathway of 3D silk fibrils grown in bulk solution.

### Two-step formation of silk multilayers

**Fig. 3.**
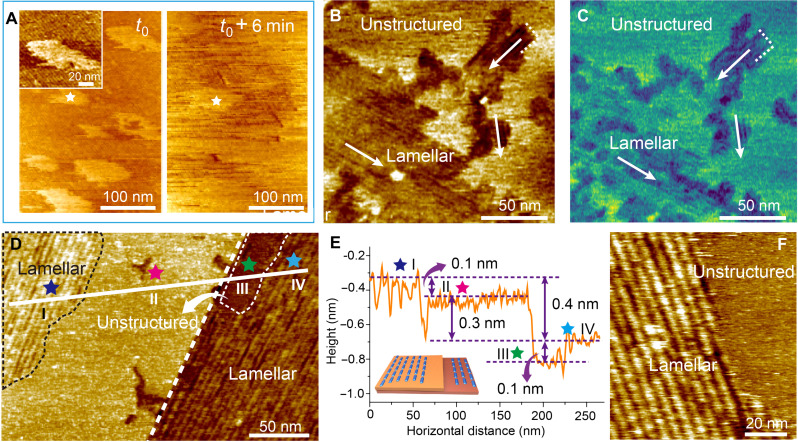
The two-step growth process of lamellar SF films. (**A**) Consecutive AFM height images of an unstructured film forming from SF solution (0.2 μg/ml). The inset shows that the unstructured film is deposited on a lamellar layer. The incubation time is 20 min. (**B** and **C**) AFM height (B) and phase images (C) during SF assembly from SF solution (1 μg/ml). The incubation time is 25 min. White arrows indicate the three directions of the lamellae. Both lamellar and unstructured domains can be easily identified in both, indicating that the domains differ in stiffness. (**D**) AFM height image showing two complete layers in which lamellar and unstructured regions coexist. The white dashed line delineates the edge of the upper layer. The black dashed boundary denotes a lamellar region in the upper layer, while the white dashed boundary denotes an unstructured region in the lower layer. The SF concentration is 0.2 μg/ml, and the incubation time is 57 min. (**E**) Height profile along the solid white line in (D). Blue, pink, green, and cyan stars denote different structural regions: lamellar (blue and cyan) and unstructured (pink and green). The inset illustrates the model of multilayer SF films with ordered and disordered regions. (**F**) High-magnification AFM height image of the boundary between lamellar and unstructured domains. The SF concentration is 0.2 μg/ml, and the incubation time is 65 min.

**Fig. 4.**
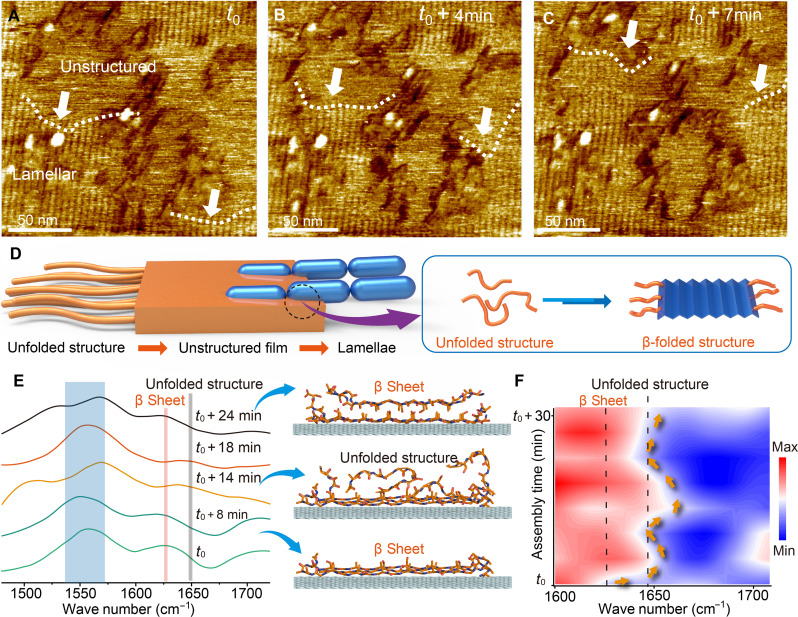
The phase transition process of lamellar SF films. (**A** to **C**) Consecutive AFM images of assembly from SF solution (1 μg/ml) showing the phase transition from unstructured to lamellar. The incubation time is 50 min. The white dashed lines mark the boundaries between the two. (**D**) Proposed model for the two-step process of the lamellar SF film formation. (**E**) In situ nano-FTIR spectra showing the change in secondary structure during assembly on graphene in high-concentration SF solution (0.5 μg/ml), along with an illustration of the process. (**F**) Corresponding color map intensity of the nano-FTIR spectra as a function of time.

When the SF concentration is further increased to ≥0.2 μg/ml, a new, unstructured layer begins to adsorb onto the underlying lamellar layer within 20 min of incubation (Fig. 3A and fig. S18). This adsorption is accompanied by the appearance of the amide I signal at 1640 to 1655 cm^−1^, implying the presence on the graphene surface of unfolded SF proteins, which are known to be mainly random coil in their unfolded state and can thus be detected by nano-FTIR through their C═O stretching vibration (fig. S19). Domains of the unstructured film coexist with lamellar domains and can be distinguished both by their topography (Fig. 3B) and their phase (Fig. 3C), with the latter generally corresponding to mechanical stiffness (*30*). The lamellae in these layers still lie along the three armchair directions of the underlying HOPG and are higher by ~0.1 nm than the unstructured film (Fig. 3, D and E) with which they form a sharp boundary (Fig. 3F and fig. S20). Time series of in situ AFM images show that the lamellar domains and the sharp boundaries are a consequence of a phase transition from the unstructured film to lamellae, which is accompanied by the increase in height (Fig. 4, A to C, and fig. S21). Hence, the unstructured film is a metastable phase consisting of unfolded SF molecules, which converts into the structured film through folding and reorganization into β sheet lamellae. This defines the second pathway of lamellar SF multilayer assembly (Fig. 4D). Moreover, these observations follow the general process of silk formation through a structural transition from random coil to β sheet conformation (*31*, *32*).

To gain insights into the structural dynamics that accompany the unstructured-to-lamellar transition, we used in situ nano-FTIR to observe the conformation state of the SF. The IR signal in the amide I region was detected but cycled back and forth between the peaks at ~1630 and ~1650 cm^−1^ associated with the β sheet and unfolded SF conformation, respectively (Fig. 4, E and F), and did so on a timescale (5 to 15 min) similar to that seen in AFM during the transition from the unstructured to lamellar film. This cyclical process facilitates the accumulation of well-ordered SF lamellar layers in the *z* direction even when the SF concentration is increased by 6× (to 3 μg/ml) (fig. S22). The intensity of the amide I peak centered at 1630 cm^−1^ is then higher than that of the amide II bands (fig. S23), demonstrating the attainment of a thick 2D SF lamellar film. Thus, these data record the cyclical process of adsorption of an unstructured film, followed by transformation to a lamellar film (schematic in Fig. 4E), and show that the unstructured film consists of unfolded SF proteins and the transition to the lamellar film is accompanied by folding into the β sheet conformation.

## DISCUSSION

**Fig. 5.**
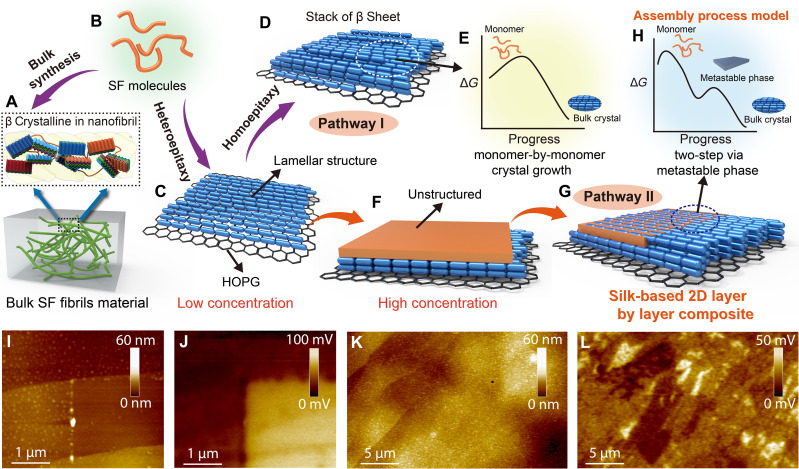
The mechanism of 2D SF assembly at the water-HOPG interface. (**A**) Depiction of bulk SF fibrils structure at mesoscopic and macroscopic scales. (**B** to **H**) Proposed scheme of 2D SF assembly at the water-HOPG interface. (**I** and **J**) AFM height image (I) and corresponding SKPM image (J) showing that the top SF film was selectively removed from the bright region in (J) through scraping to reveal the underlying HOPG where the surface potential is significantly higher. (**K** and **L**) AFM height image (K) and corresponding SKPM image (L) of SF film on HOPG.

The above findings show that, in contrast to bulk SF fibrils, which are both mesoscopically and macroscopically disordered (Fig. 5A), SF self-assembles in an epitaxial manner into well-ordered 2D lamellar films on HOPG, with the building units of the film exhibiting the same secondary structure as the β sheet crystallites in the bulk form, in which the β sheets stack face to face. While two assembly pathways arise because of the competition between the adsorption rate of unfolded SF molecules and the folding and assembly into the lamellae, the outcome remains a well-ordered 2D lamellar film (Fig. 5, B to H).

The progression of growth regime from direct formation of the ordered monolayer to direct formation of multilayers to multilayer formation via two-step mechanism is accompanied by changes in the rate-limiting processes. At low concentrations (0.01 to 0.08 μg/ml), where a lamellar monolayer forms directly, the main control is the SF concentration. As discussed above, the longitudinal growth rate in linearly dependent on the concentration. Moreover, the incubation time for the appearance of the first SF lamellae scales approximately inversely with concentration. For instance, the incubation time at ultralow concentrations (<0.01 μg/ml) is ~70 min, but this is reduced to only 10 min when the concentration is increased to 0.05 μg/ml. While the value of the kinetic coefficient suggests that SF desolvation is the rate-limiting process for the longitudinal growth, we speculate that the time needed for SF folding on the HOPG surface into the β sheet structure controls the initial incubation time.

When the SF concentration is increased to the range in which multiple lamellar layers can form through a direct pathway (≥0.1 μg/ml), SF concentration remains the external control on rate. However, the attachment process is clearly slowed down because the longitudinal growth rate at 0.1 μg/ml is about 20 nm/min (Fig. 2), whereas an extrapolation of the data for the first monolayer in fig. S8 gives 71 nm/min. Moreover, lamellae of the second layer form only after some 50 to 60 min of incubation time, whereas, at these concentrations, the first monolayer forms in minutes—before imaging even begins. These differences indicate that the interaction of SF monomers with the underlying SF monolayer inhibits either the folding of SF molecules as they attach to the lamellae or possibly surface diffusion of adsorbed SF monomers to the forming lamellae. However, our AFM data alone are insufficient to justify a definitive conclusion.

Once the concentration reaches the regime in which multilayer growth involves a two-step process of unstructured film formation followed by transformation to ordered lamellae, the folding transition is clearly rate limiting. Three observations support this conclusion. First, the growth front between the unstructured and structured film advances at only 8.6 nm/min at an SF concentration of 1.0 μg/ml, a concentration at which an extrapolation of the data in fig. S8 would give a speed of over 760 nm/min. Second, concentration has little or no effect on the growth rate of formed lamellae; rather, it is completely determined by the transformation rate; for example, within error, the speed is the same even when the concentration is increased threefold to 3 μg/ml (fig. S24). The fact that the growth of lamellae within the unstructured film is much slower than would be expected at these concentrations based on the growth rates of lamella that form directly, either in the first monolayer or the in multilayer, indicates that the transformation is a cooperative phenomenon. Despite the slow transformation rates, initial formation of the unstructured film is rapid, with unstructured domains appearing within 20 min at 0.2 μg/ml and, at the highest concentrations used here, completely covering the surface before the appearance of any ordered lamellar domains.

Given the high degree of crystalline packing in these lamellar films, in analogy to films of conjugated polymers, we might expect them to exhibit strong orbital overlap (*33*), which has been shown to improve the electronic performance of film-based devices, such as perovskite solar cells (*34*). Heteroepitaxial assembly of 2D SF films also occurs on other 2D vdW surfaces, such as MoS_2_ (fig. S25), although not on the hydrophilic surface of mica (fig. S26). Consequently, this 2D phase of SF may be a useful construct for modulating the electronic properties of vdW materials in general. Surface potential measurements using scanning Kelvin probe microscopy (SKPM) on HOPG demonstrate a 60-mV decrease in potential following SF assembly (Fig. 5, I and J). Such a decrease has been shown to significantly reduce the barrier to metal ion migration and increase the transport rate of charged particles in protein films (*5*). In addition, because differences in SF film thickness lead to variations in surface potential (Fig. 5, K and L), systematic design of multilayers can enable programmable modulation of electronic properties. Moreover, although domains with orientations distinct from the dominant armchair direction are unstable in the presence of the latter and are eliminated through coarsening (fig. S27), the fact that these domains still form suggests sufficiently weak film-substrate binding that a fully formed, cross-linked film could be twisted relative to the underlying vdW substrate to achieve additional modulation of the electronic properties, just as interlayer twisting of the vdW materials has been used for this purpose (*35*, *36*). Together, these results and considerations suggest that the ability to create highly ordered 2D SF layers on multiple vdW materials provides an unexplored strategy for both extending and improving the performance of silk in electronic and optical applications.

## MATERIALS AND METHODS

### Material

SF was regenerated from the cocoons of domesticated *Bombyx mori* silkworms (Guangxi Sericulture Technology Co. Ltd.) as previously described (*5*). The fresh SF solution was injected into liquid nitrogen through a syringe pump to form small globules and then freeze-dried for 3 days. The dried SF globules were stored at −20°C before further usage. Sodium bicarbonate (NaHCO_3_) and isopropanol were obtained from Sinopharm Chemical Reagent. Lithium bromide (LiBr) was provided by Aladdin Industrial Corporation. HOPG (HOPG grade ZYB, 0.8°, 10 mm × 10 mm × 1 mm) was purchased from Ted Pella Inc.

### In situ AFM characterization

SF powders were mixed with 10 ml of nuclease-free water (Ambion, US) and purified with 0.2-μm filter to remove aggregate and insoluble SF. Eighty microliters of SF solution was added on top of a freshly cleaved HOPG surface at room temperature. The 2D assembly requires a suitable concentration, which should be below 30 μg/ml to avoid triggering the formation of 3D SF nanofibrils (fig. S28). All in situ images were captured under tapping mode with a Cypher ES AFM (Asylum Research). Silicon probes (SNL-C; *k*, 0.24 N/m; tip radius, 2 nm; Bruker) were used for all images except those in Fig. 5, which were captured using platinum-coated AFM cantilevers (Multi75E, BudgetSensors). AFM height and phase images of SF were collected at scan frequencies of 1.5 Hz, and the AFM image of the HOPG lattice was collected at scan frequencies of 7 to 9 Hz. AFM height images were analyzed using Scanning Probe Image Processor (SPIP) (Image Metrology, Denmark); AFM phase images were processed by Gwyddion SPM data analysis software.

### In situ nano-FTIR characterization

The present study used the technique of in situ nano-FTIR characterization, building upon the previous research (*27*). The measurements using nano-FTIR were conducted at beamline 2.4 of the Advanced Light Source, located at the Lawrence Berkeley National Laboratory. During the experimental setup, IR light was carefully directed onto the Pt-coated AFM tip within a neaSNOM (near-field scanning optical microscopy) system. Detailed information regarding the fabrication of the graphene membrane can be found in prior publications. For the experimental procedure, the SF solution was enclosed within the graphene liquid cell immediately before the commencement of the experiment, denoted as time 0. To establish reference spectra, samples with flat spectral responses were used, including either Au-coated Si or graphene on the Au-coated SiN membrane. Tapping-mode operation was used, operating at the fundamental resonance frequency of the cantilever (ranging from 250 to 350 kHz) with a free oscillation amplitude varying between 70 and 90 nm. The amplitude set point was maintained at approximately 80%. To eliminate the far-field nonlocal scattered background, the scattered near-field signal was extracted using a lock-in amplifier that was tuned to the second and higher harmonics of the cantilever oscillation.

### In situ circular dichroism spectrum

The circular dichroism spectrum of SF (0.2 mg/ml) was collected on a circular dichroism spectropolarimeter (Applied Photophysics Ltd.) at room temperature lasted 80 hours.

### Attenuated total reflection–Fourier transform infrared

The SF solution (0.27 mg/ml) was cast on HOPG and incubated for 1 hour, then remove the water, and dried under fume hood. The ATR-FTIR measurements were conducted by an FTIR PerkinElmer Frontier with a diamond crystal ATR module. The spectra were collected with a resolution of 4 cm^−1^ in the spectral range from 4000 to 400 cm^−1^ averaging over 64 scans and normalized to the light intensity.

### Scanning Kelvin probe microscopy

The SF solution (0.1 μg/ml) was casted on HOPG, incubated for 1 hour, then removed the water, and dried under fume hood. Kelvin probe force microscopy was carried out using Cypher S AFM (Asylum Research, CA) in the air with platinum-coated AFM cantilevers (Multi75E, BudgetSensors) under lift or dual-pass mode. In SKPM measurements, the sample was grounded. The offline data process was done with SPIP software (Image Metrology, Denmark).

### MD simulations

MD simulations were run with the GROMACS 2021.34 engine and PLUMED 2.7 plugin, using the CHARMM36m and Interface force fields as well as the TIP3P water model. The Interface force field has been parameterized as an extension for CHARMM36m. As a result, it is both compatible for use with proteins and capable of comparing between different surface species (*37*). All simulations were run at pH 7 with charged termini. The Parrinello-Rahman barostat was used for simulations in the NPT (isothermal-isobaric) ensemble, with compressibility set to zero in the *x*-*y* direction, and a time constant of 2 ps. Temperature was coupled using a Noose-Hoover extended ensemble with a time constant of 2 ps. Because simulations only involved near-surface and bound proteins, a full 3D periodic treatment was used for all systems. All systems were energy minimized before equilibration in the NVT (canonical) and NPT ensembles. The LINCS algorithm was used to restrain hydrogen bonds. Production simulations were run in the NVT ensemble. Folded SF protein structures were generated with PyMOL using the structure builder to generate the β strand linker motif [(GAGAGS)_2_GAAS], which was subsequently copied and bonded to generate the full protein. Surface structures were generated with CHARMM-GUI. To replicate experimental conditions, all graphite surfaces were composed of five layers. All simulations of 2D SF on HOPG assumed a folded β sheet structure, as determined from the nano-FTIR data (Fig. 2, F and G). Analysis was performed with the NumPy, MDAnalysis, and MDTraj Python libraries, and figures were generated with Matplotlib, PyMOL, and VMD. Simulations were run on 2080ti graphics processing units on the University of Washington Klone cluster. All input files and scripts necessary to replicate the simulations performed in this study are available for download at the following archived repository: zenodo.org/records/11246664. The distribution of angles between C═O bonds and graphite was calculated by defining vectors along C═O bonds and calculating the angle between these vectors and the *z*-normal vector with the following equation

$$\theta ={\text{cos}}^{-1}\frac{a\cdot b}{\left|a\right|\left|b\right|}$$

where *a* is the vector formed by C═O bonds and *b* is the *Z*-normal vector. Because the *Z*-normal vector is perpendicular to the *xy* plane, the resulting angles can easily be converted to angles between C═O bonds and the *xy* plane by subtracting 90°. The *xy* plane was chosen as a representation of graphite because the surface is perfectly flat in the *xy* plane during simulation. Simulation results on HOPG were validated by comparing SF binding and secondary structure stabilities to those of SF on mica, as this surface was experimentally shown to not support lamellae growth (figs. S26 and S29) and acts as a negative control.
