# Supplementary material for: Two-dimensional silk
Source: Sci Adv. 2024 Sep 18;10(38):eado4142. doi: 10.1126/sciadv.ado4142 (PMC11409968; doi:10.1126/sciadv.ado4142)
Supplement: Supplementary file 1 — Supplementary Text Figs. S1 to S30 Tables S1 and S2 [file sciadv.ado4142_sm.pdf]

Science Advances
AAAS

Supplementary Materials for

Two-dimensional silk

Chenyang Shi *et al.*

Corresponding author: James J. De Yoreo, james.deyoreo@pnnl.gov; Shuai Zhang, zhangs71@uw.edu; Xiang Yang Liu, liuxy@xmu.edu.cn

Sci. Adv. **10**, eado4142 (2024)
DOI: 10.1126/sciadv.ado4142

This PDF file includes:

Supplementary Text
Figs. S1 to S30
Tables S1 and S2

Supplementary Text

We tested orientation preferences of SF β-sheet on HOPG as a validation of the force fields (fig. S5). SF was placed ~1 nm above the surface, and the backbone was restrained using the PLUMED ANTIBETARMSD collected variable. Given the sequence of SF ($(GAGAGS)_2GAAS$) and its β-sheet structure, it has two distinct sides: one populated by glycine sidechains, and one populated by alanine and serine sidechains. We found that SF only remains folded on HOPG when bound with alanine sidechains facing bulk solvent – when facing the surface, waters are able to intercalate and the protein unfolds (Fig. S30). To that end, all simulations of SF were performed such that alanine sidechains faced bulk solution (*20*). When bound with sidechains facing the solution, SF was stable over 500 ns (Fig. S30A, B). Three initial orientations were tested: one in which the SF β-sheet was aligned with the armchair edge, one in which it was aligned with the zigzag edge, and an intermediate orientation. The SF β-sheet bound to the surface completely within 5 ns in all three trials. The SF β-sheet displayed a strong preference for the armchair orientation. Both the intermediate and zigzag orientations aligned to the armchair edge during binding and stayed at this orientation for the remainder of the 100 ns simulation. The armchair orientation did not rotate during binding. Note that these simulations do not provide any mechanistic information and are simply a validation that the force field is able to reproduce orientational preference.

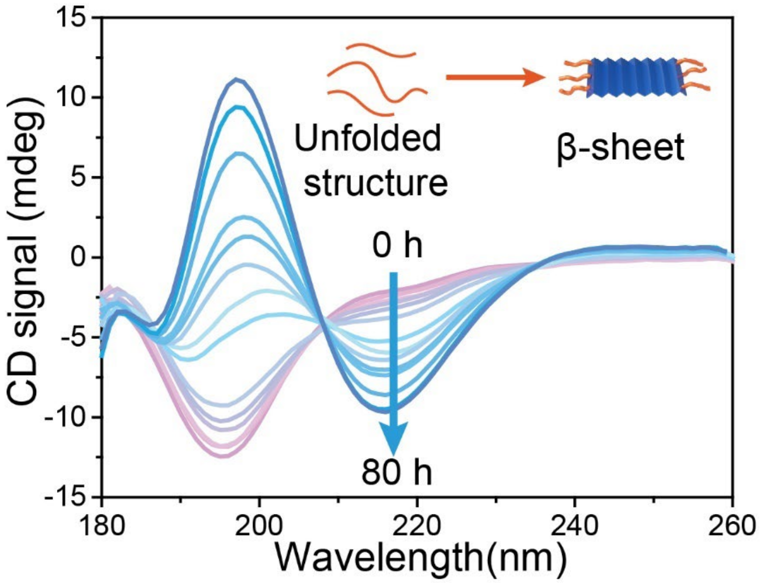

Fig. S1. Circular dichroism (CD) spectrum of SF solution. The observation of a negative peak in the region around 190–200 nm indicates that the freshly prepared SF solution primarily consists of unfolded structures, predominantly in the form of random coils. Over time, these unfolded structures undergo a gradual transformation into β-sheets, which represents a natural phenomenon in the nucleation and growth process of silk materials. Note: β-sheets shows a negative band at 218 nm and a positive one at 196 nm.

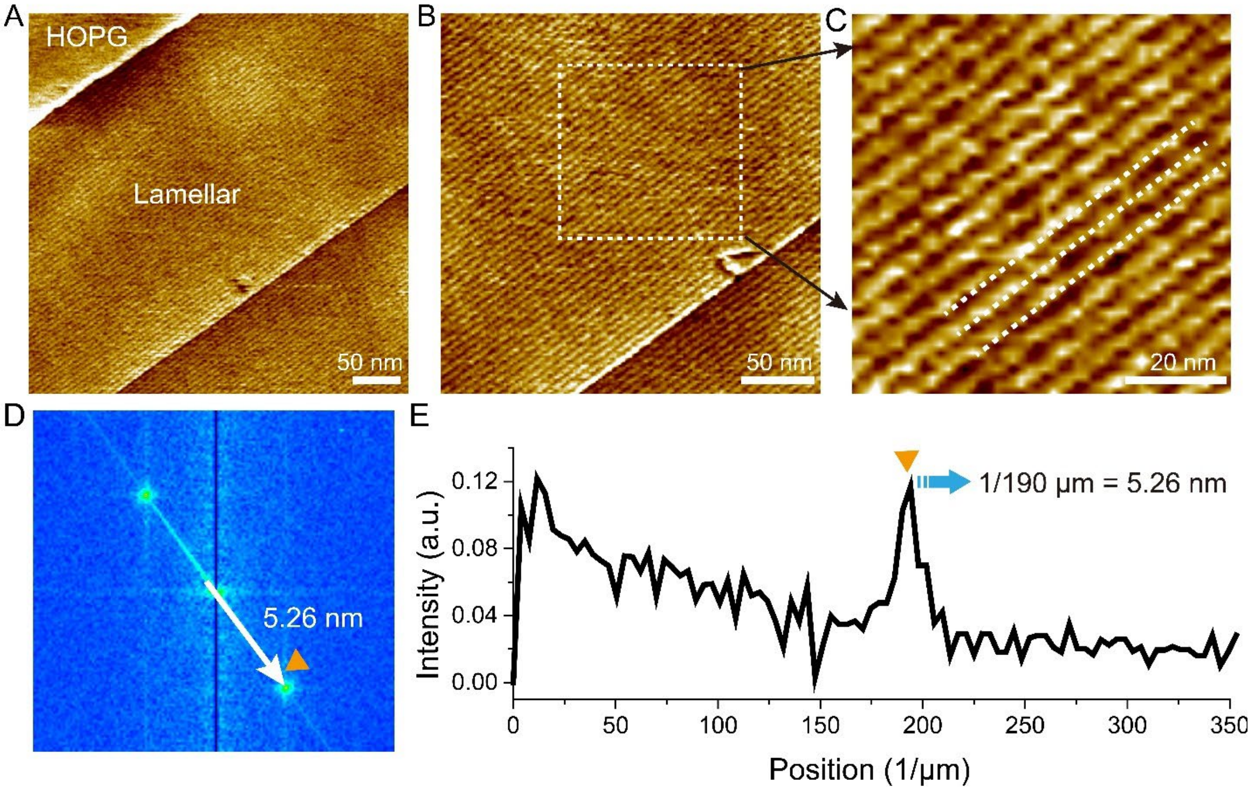

Fig. S2. Periodic width of lamellar structure of SF. (**A** to **C**) Topography map of different scale. (**D**) FFT image. (**E**) 1D Fourier profile. (The concentration of SF is 0.05 μg/mL)

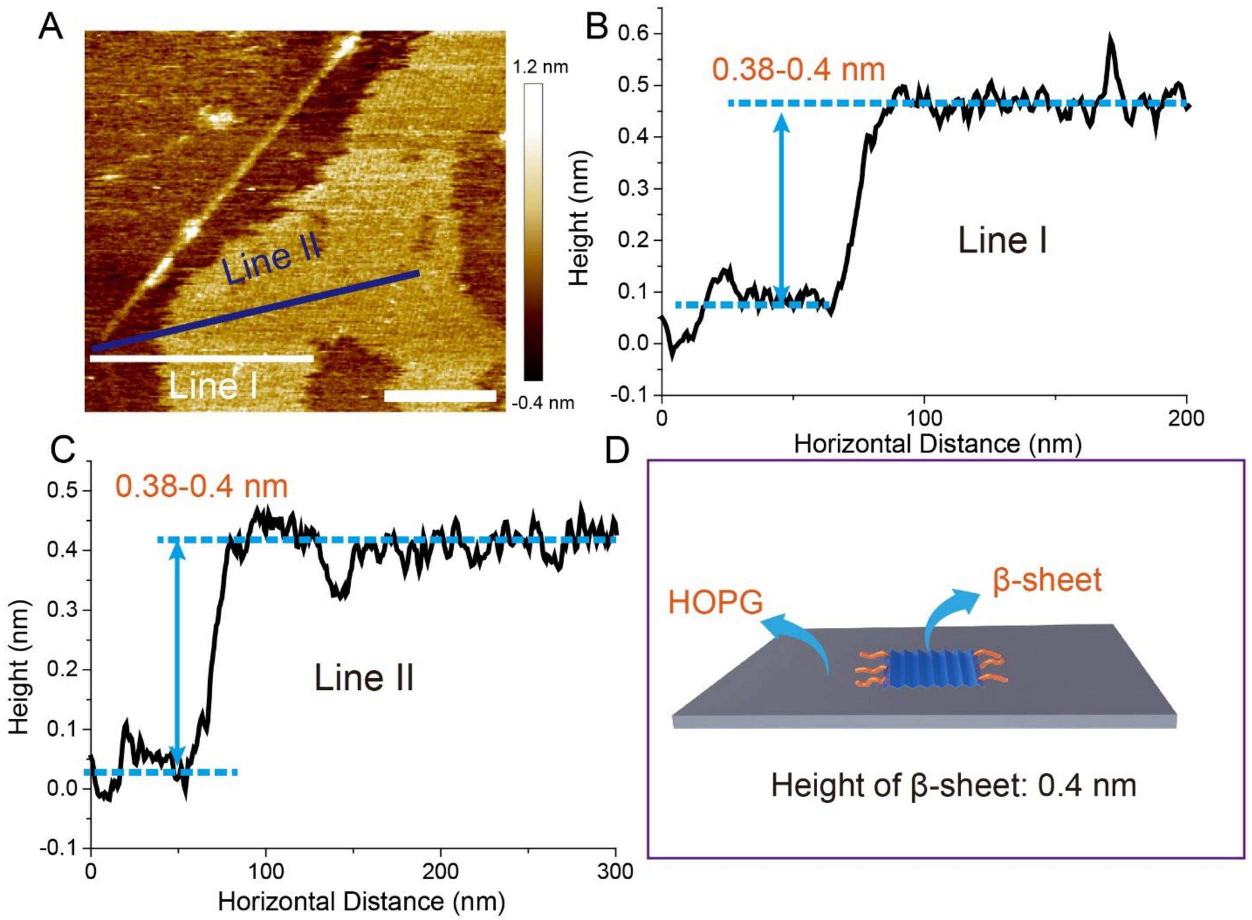

Fig. S3. Height information for lamellar structure of SF. (**A**) Topography map, scale bar is 100 nm. (**B**) Height profile for line 1. (**C**) Height profile for line 2. (**D**) Schematic drawing of β-sheets structure on HOPG. (The concentration of SF is 0.05 μg/mL)

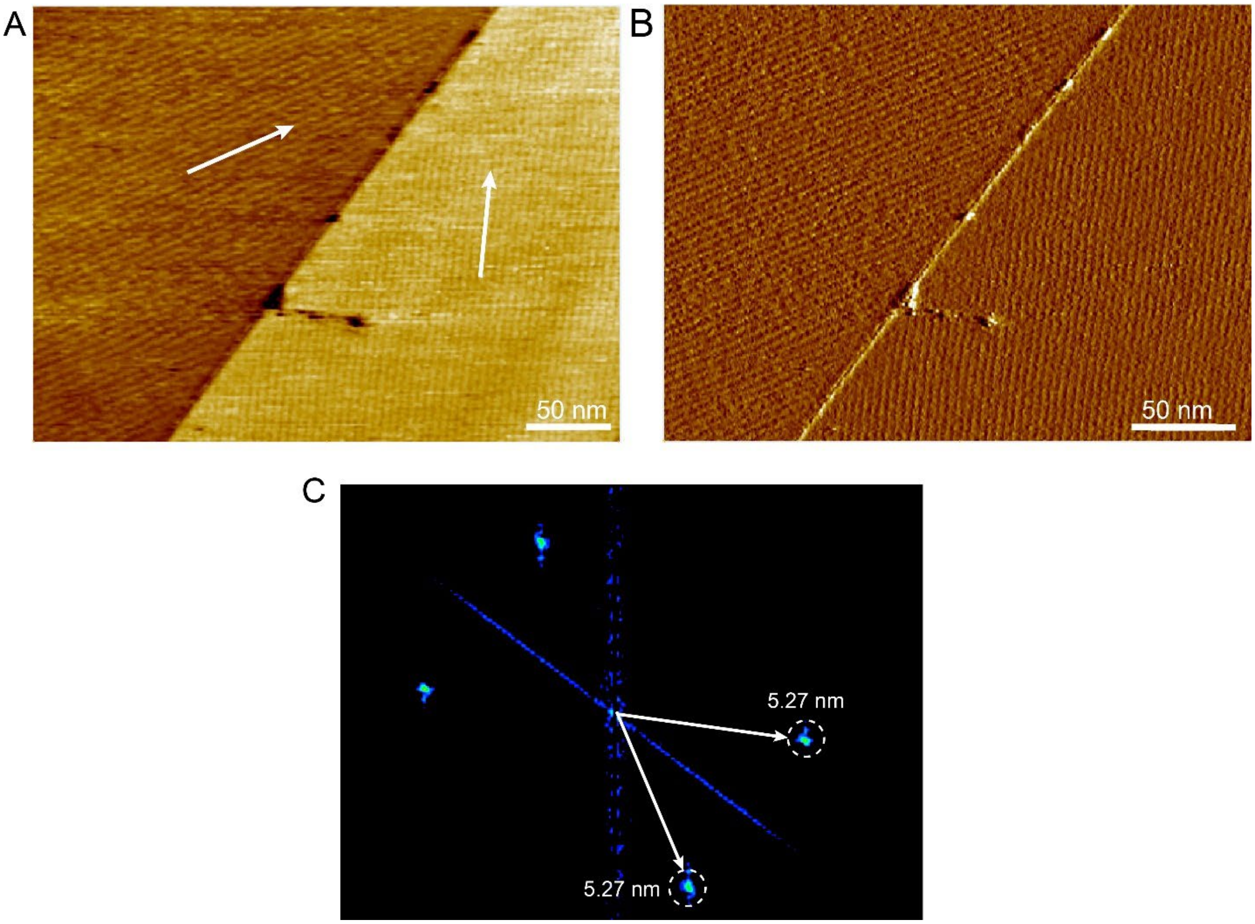

Fig. S4. The orientation of SF lamellae. SF assembled into lamellae which formed at an angle of 120° to each other, indicating aligned along three equivalent directions on HOPG. (**A**) Height image. (**B**) Amplitude image. (**C**) Corresponded FFT image.

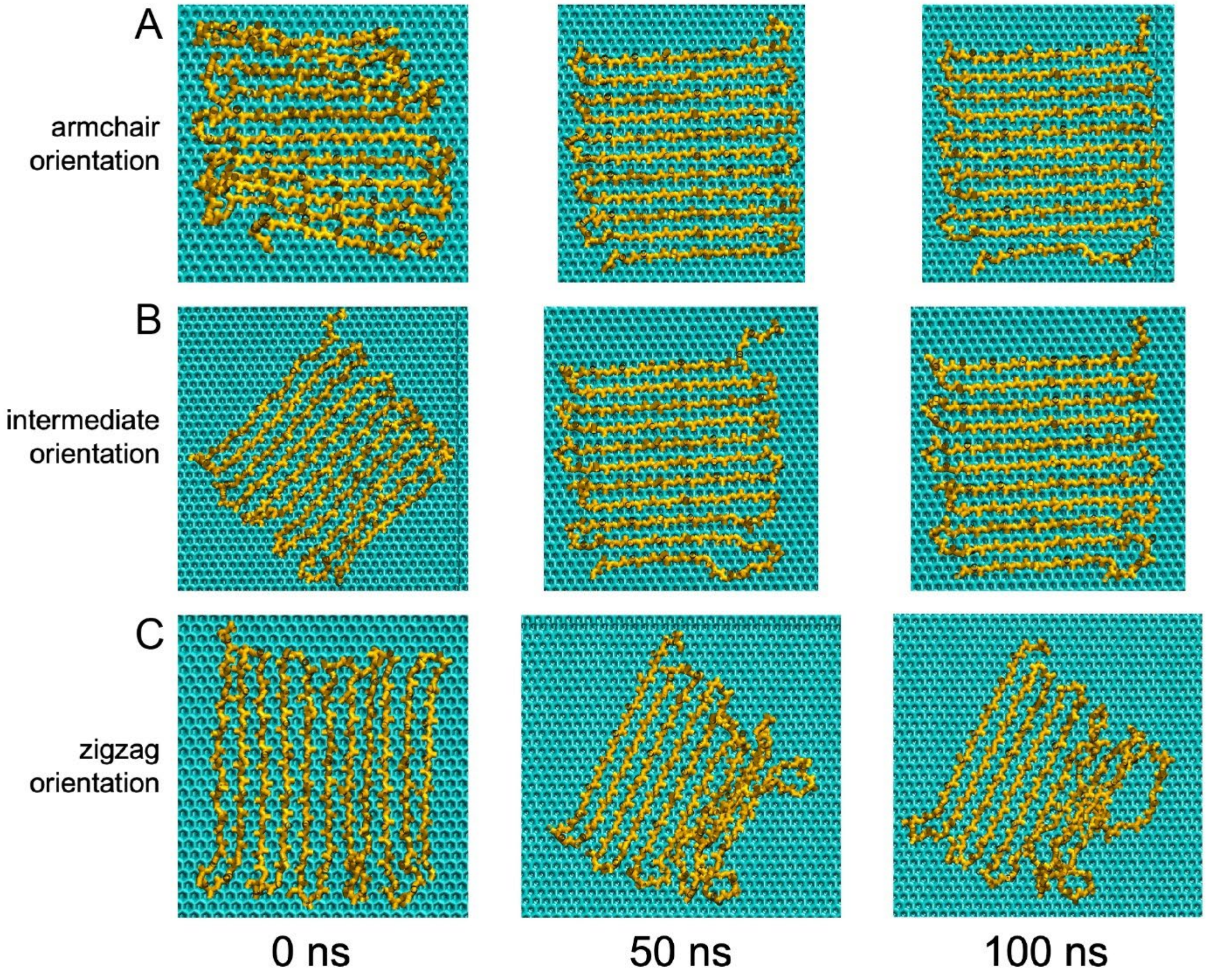

Fig. S5. Simulations of SF β-sheets binding to HOPG. SF β-sheet was placed ~1 nm above graphite and allowed to bind. The protein initially aligned along the armchair edge. (**A**) did not rotate. However, the proteins aligned to the intermediate and zigzag orientations, (**B** and **C**) rotated to align to the armchair edge during binding, and remained at this orientation throughout the simulation.

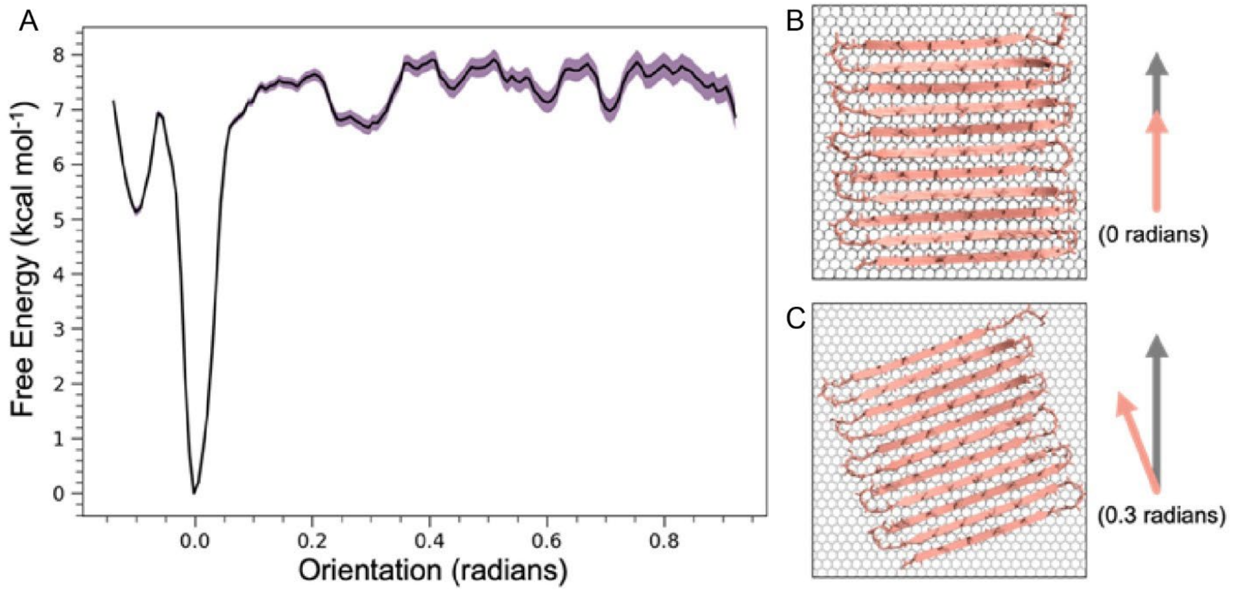

Fig. S6. Energetics of SF on HOPG. (**A**) Free energy of a single SF protein as a function of orientation to the armchair edge of graphite with error bars drawn. (**B**) SF orientation at 0 radians. This is the most stable configuration and corresponds to protein alignment along the armchair edge of graphite. (**C**) SF at 0.3 rad, a metastable state in which the protein is more closely aligned to the zigzag edge of graphite. Free energy surface of SF orientation on HOPG, with a global minimum when SF is aligned along the armchair edge. This conformation is stabilized by backbone carbon-surface interactions. Figure used with permission from Zorman et al (*20*).

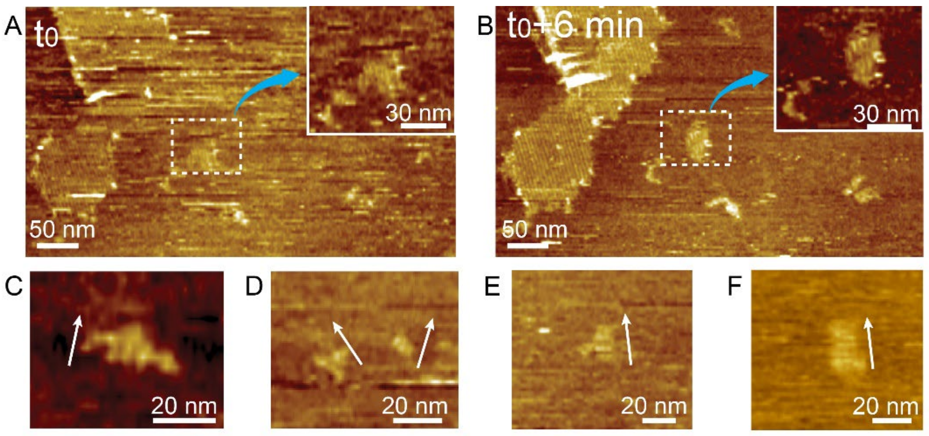

Fig. S7. In situ AFM height images showing that the SF molecules attach to the HOPG surface and grow directly into lamellae. (**A**, **B**) Consecutive AFM height images of SF lamellae. The inset showcases the corresponding magnified images within white dashed boxes. (**C**-**F**) AFM height images show formation of SF lamellae islands. SF concentration: 0.05 µg/mL, incubation time t_0 is 10 min. The arrows in image C-F represent the orientation direction.

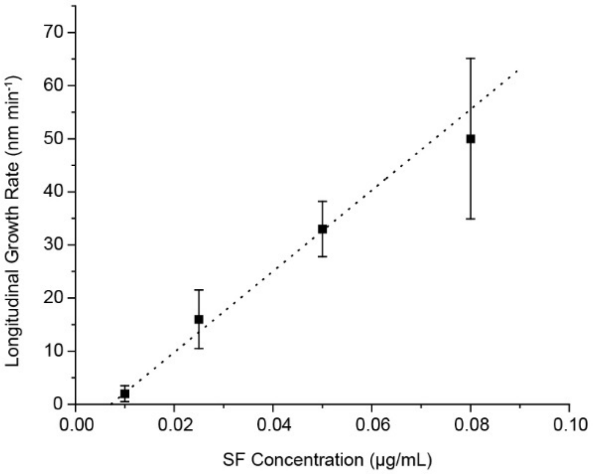

Fig. S8. Longitudinal growth rate of monolayer lamellae. To obtain a more accurate growth rate, 20 lamellar rows in each concentration were measured and calculated the average value.

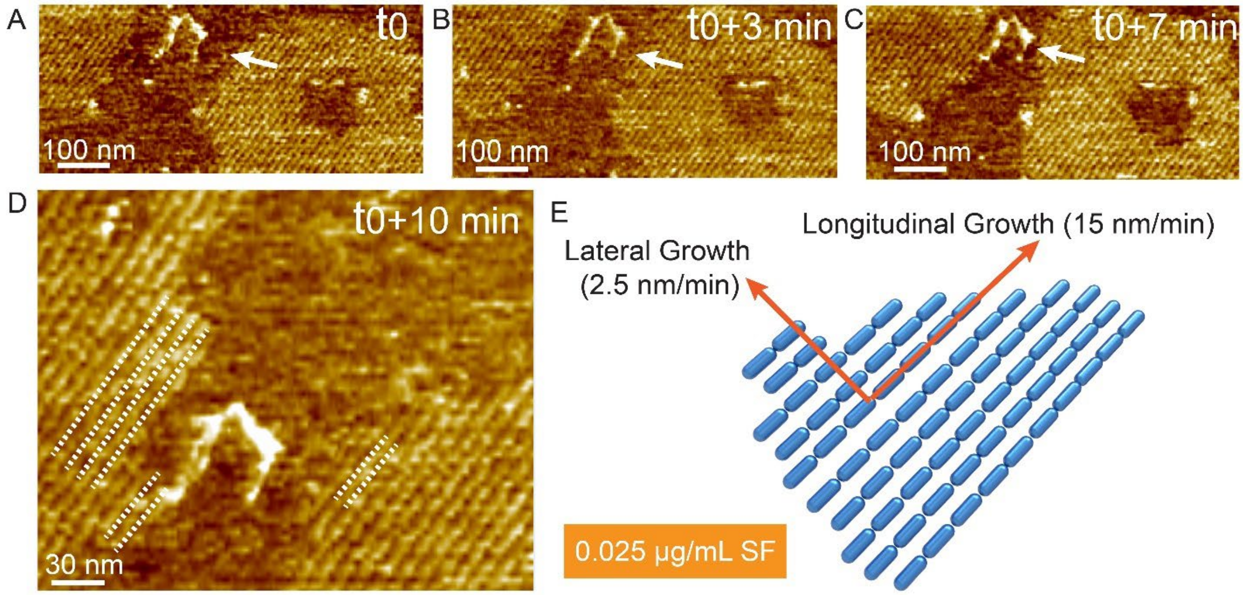

Fig. S9. Nucleation of new lamellae adjacent to existing rows. (**A-D**) Consecutive AFM height images of the lamellar structure forming from 0.025 μg/mL SF solution. (**E**) Proposed model for the SF lamellar growth. The white arrow represents the growth direction of SF lamellae.

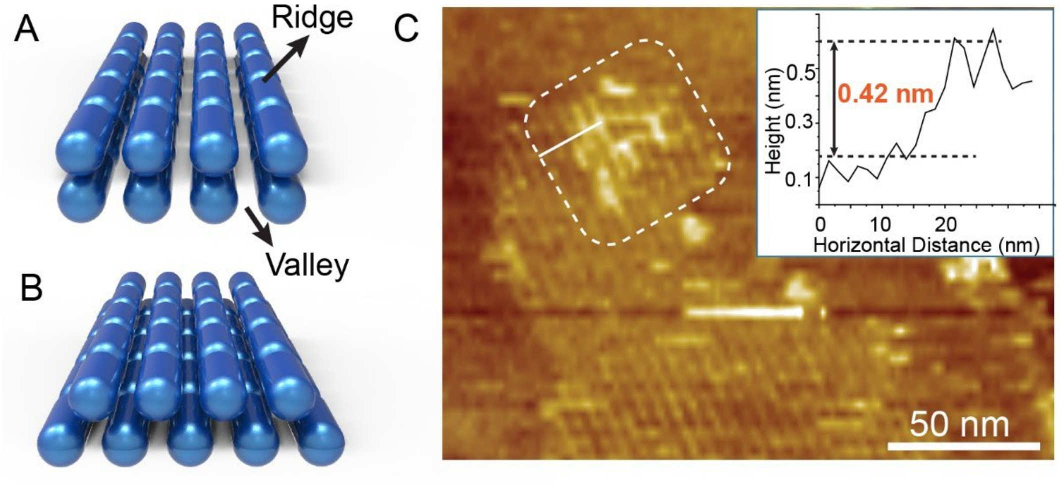

Fig. S10. The co-aligned growth of lamellae. (**A** and **B**) The lamella model of co-align growth, the upper lamella grew along the "ridge" of the underneath lamellar structure (A) rather than lateral dislocation to the "valley" of the underneath lamellar structure (B), indicating SF β-strands precisely matching in vertical orientation induced by intermolecular interaction inside groups. (**C**) A high-magnification AFM image of the multilayer lamella structure. The white dashed circle highlights the detail of the stacked parallel lamella. Inset is the height line-profile along the white line in the white dashed circle.

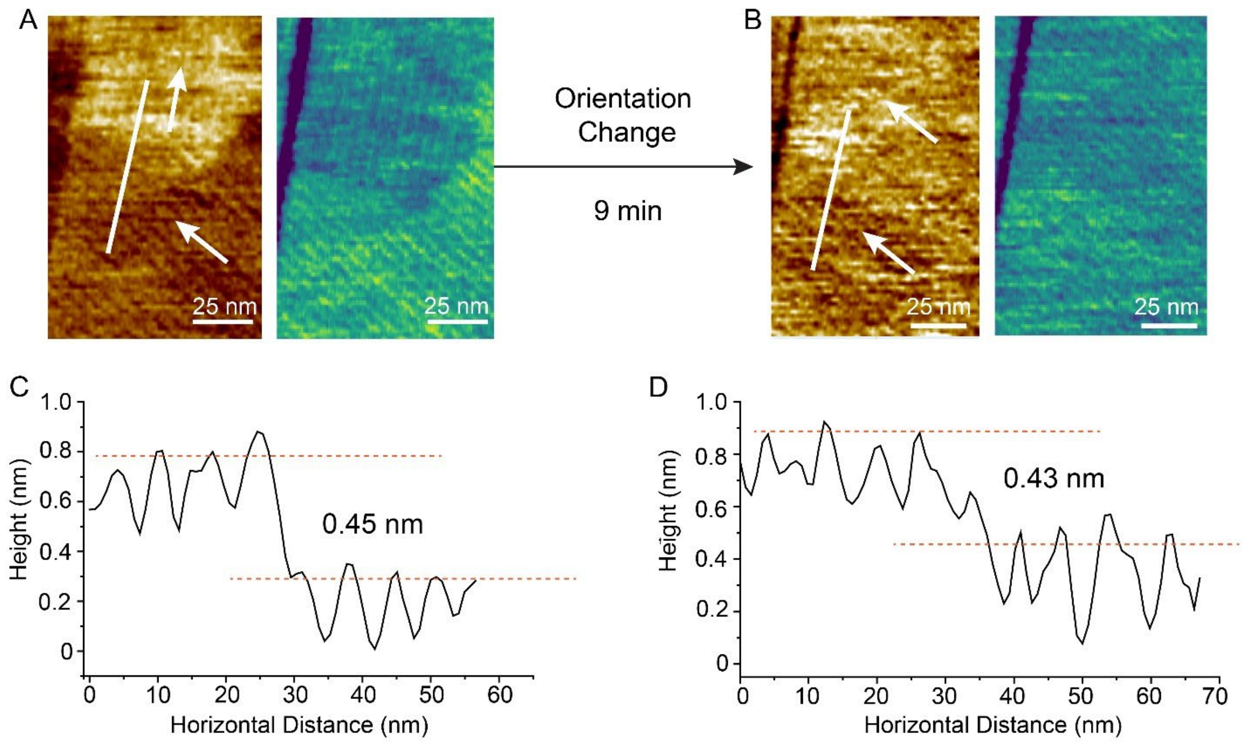

Fig. S11. The transition from crossed lamellae to co-aligned lamellae. The transition from crossed lamellae to co-aligned lamellae. (**A**) The topography AFM image (left panel) and phase AFM image (right panel) of two types of lamellae stacking before orientation changing. (**B**) The topography AFM image (left panel) and phase AFM image (right panel) of lamellae after orientation changing. (**C**) Height profile along the solid white line in (A). (**D**) Height profile along the solid white line in (B). The white arrows represent the lamellae orientations. The difference in height between the crossed lamellae is maintained during and after the transition to co-aligned lamellae.

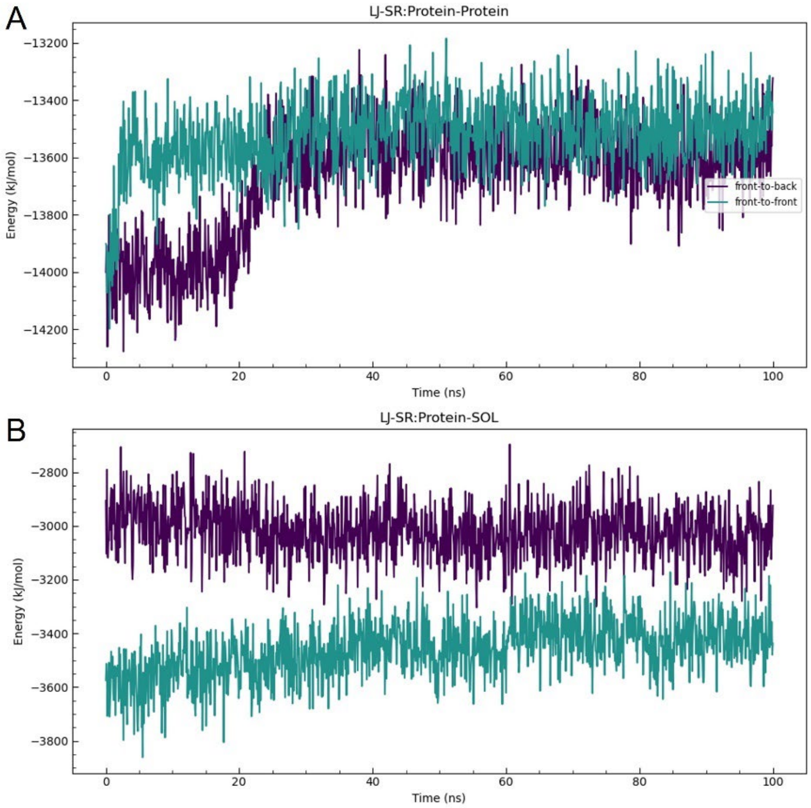

Fig. S12. Short-range Lennard Jones (LJ) interactions of bilayer SF. (**A**) Protein-protein interactions are similar for both the front-to-back configuration. (**B**) The front-to-front configuration had more favorable protein-solvent interactions. Purple: front to back, cyan: front to front.

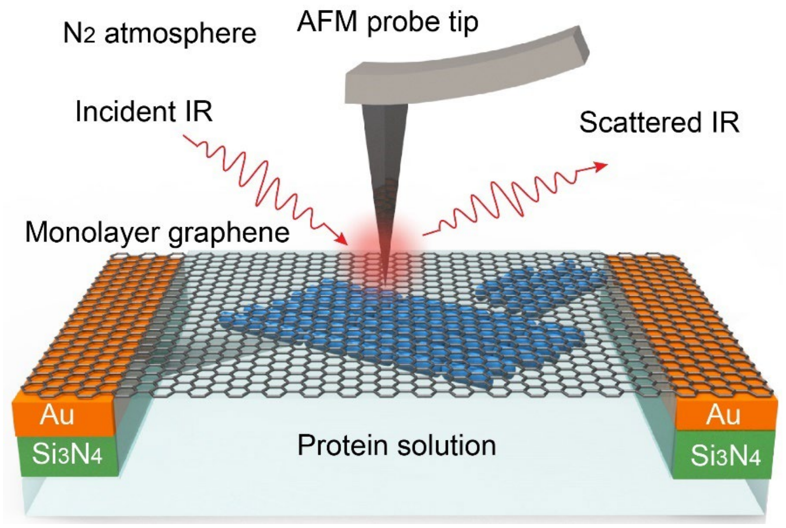

Fig. S13. Schematic of the nano-FTIR experiment. The monolayer graphene separates the air and protein solution.

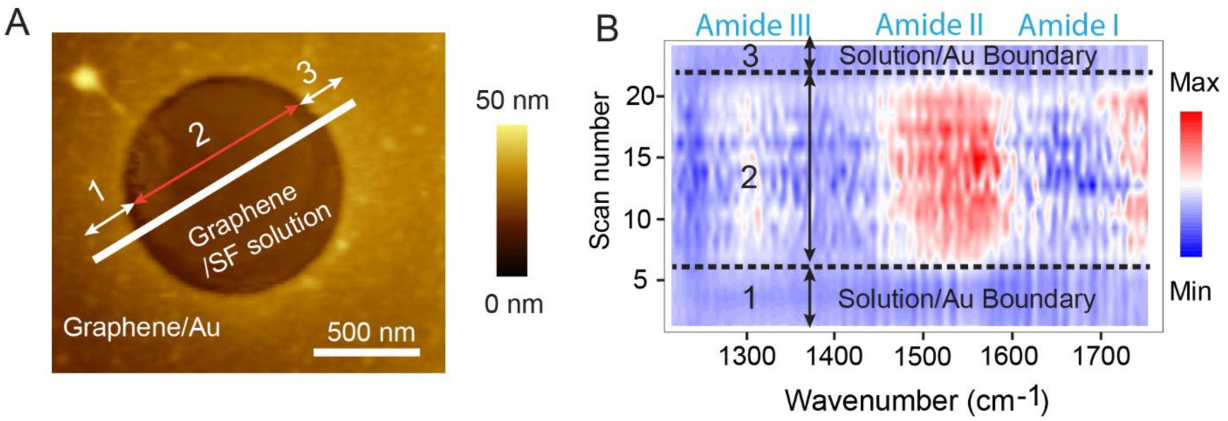

Fig. S14. Spatially resolved chemical mapping of SF assemblies. (**A**) Images of the total scattered optical amplitude of suspended graphene and surrounding area in the cell filled with SF (0.1 μg/mL). (**B**) Corresponding color map representations of the nano-FTIR spectral intensities of the amide I, II, and III mode regions of the SF. The nano-FTIR profiles were acquired at positions along the white line in image (A). The number 1,2 and 3 represent the location of graphene/Au, graphene/SF solution and graphene/Au, respectively.

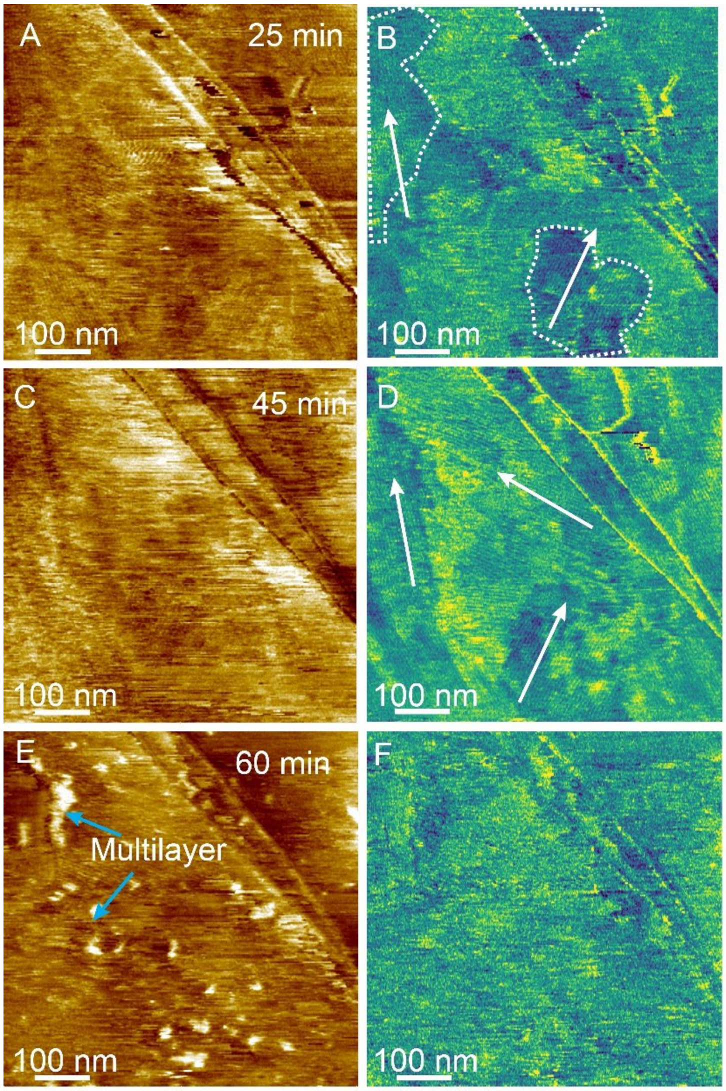

Fig. S15. The growth process of multilayer SF lamellae. (**A** and **B**) The height AFM image (A) and phase AFM image (B) of monolayer lamellae on the HOPG surface. (**C** and **D**) The height AFM image (C) and phase AFM image (D) of monolayer lamellae fully covered the HOPG surface. (**E** and **F**) The height AFM image (E) and phase AFM image (F) of multilayer lamellae.

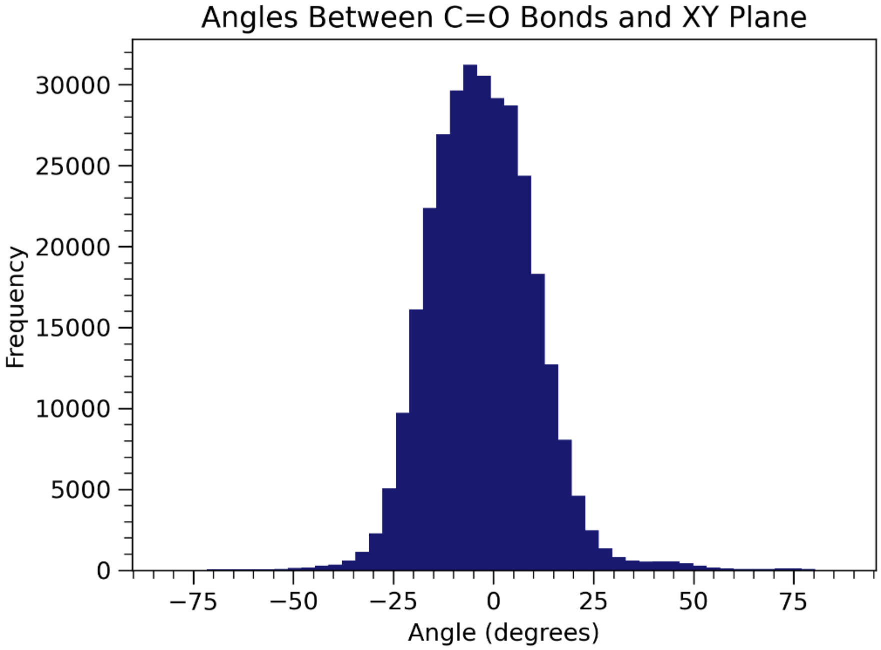

Fig. S16. Distribution of angles between vector defined by protein backbone C=O bonds and the XY plane. The XY plane was chosen as a proxy for graphite as the surface is exactly flat along the XY plane in the simulation. Angles were calculated only for non-turn residues, as turns are lifted and therefore preclude C=O bonds from lying flat. The distribution spread is likely a result of thermal fluctuations in atomic positions.

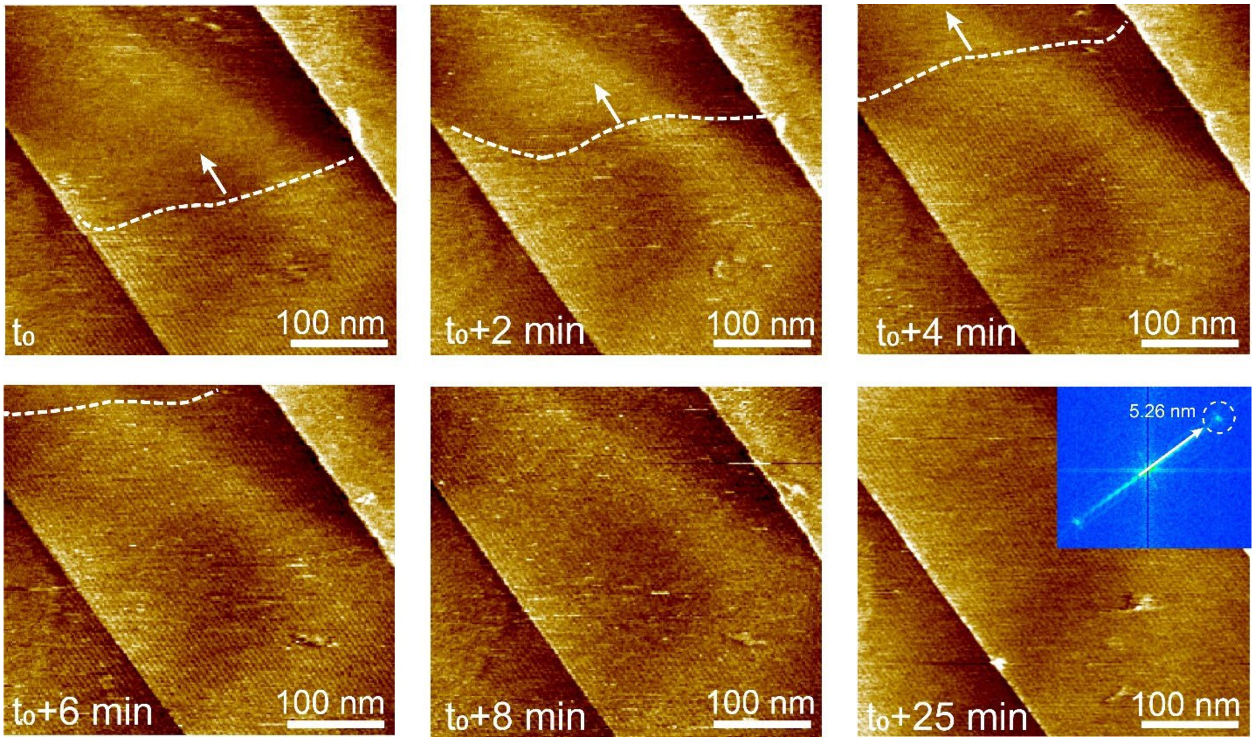

Fig. S17. AFM images of SF self-assembly at different growth times. The illustration in t_0+25 min image is the corresponding FFT image, SF concentration: 0.05 μg/mL, incubation time t_0: 31 min.

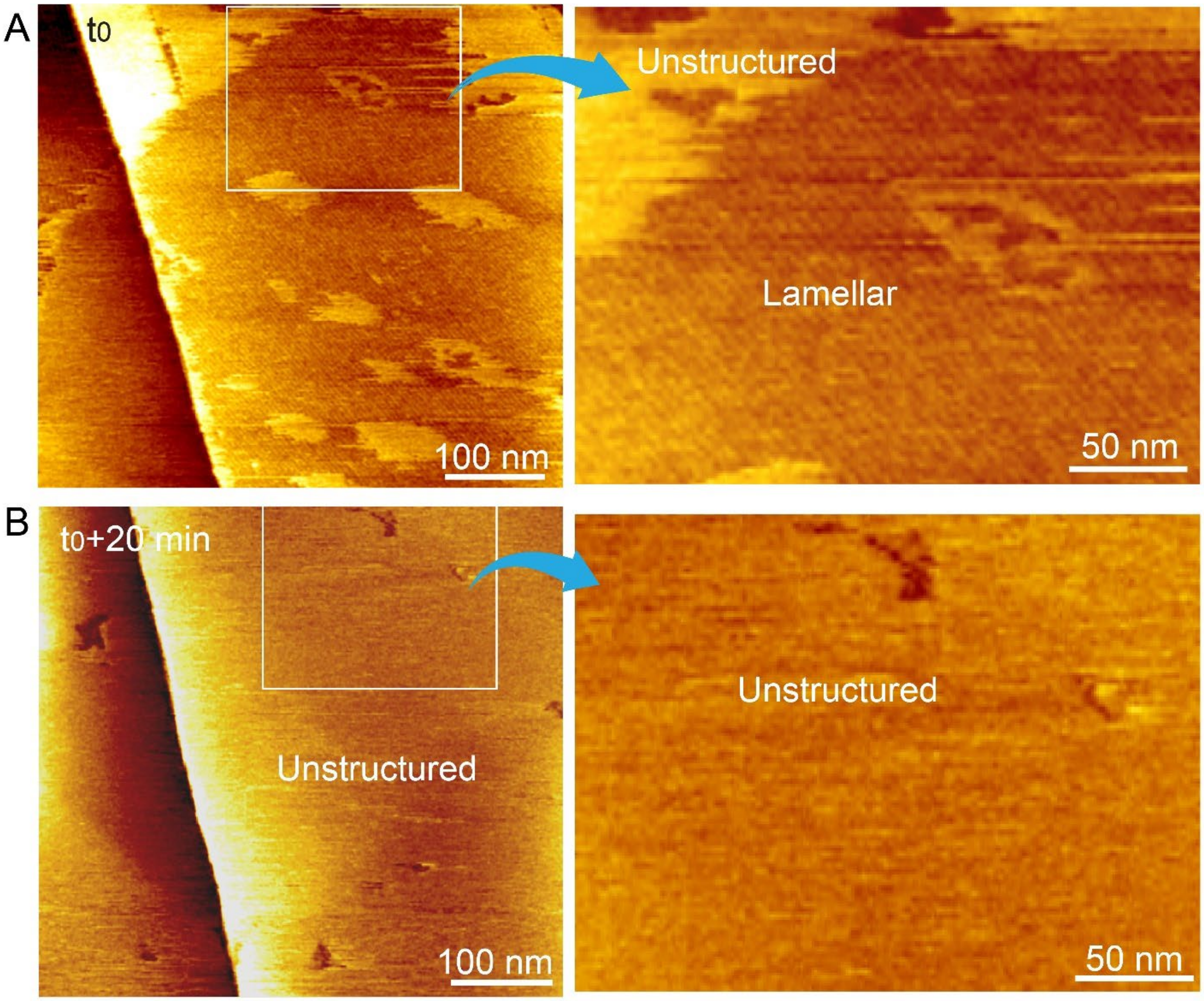

Fig. S18. Consecutive AFM height images of an unstructured film forming from 0.2 µg/mL SF solution. (**A**) The height AFM image at t_0 (left panel) and the higher magnification image from A (right panel). (**B**) The height AFM image after 20 mins (left panel) and the higher magnification image from A (right panel).

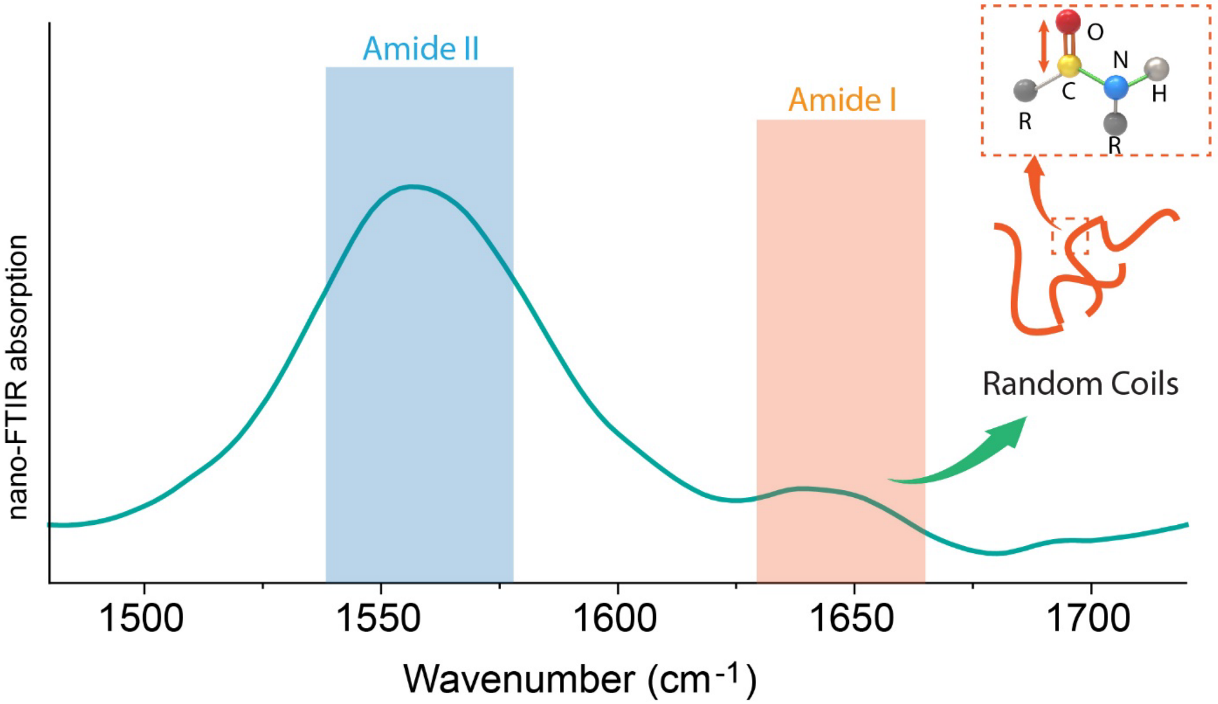

Fig. S19. Nano-FTIR spectra of an assembled SF structure in 0.5 μg/mL SF solution. The detection of the amide I signal during assembly above a concentration of 0.5 μg/mL SF solution serves as evidence for the presence of an unfolded structure (such as random coil and α-helix) on the surface.

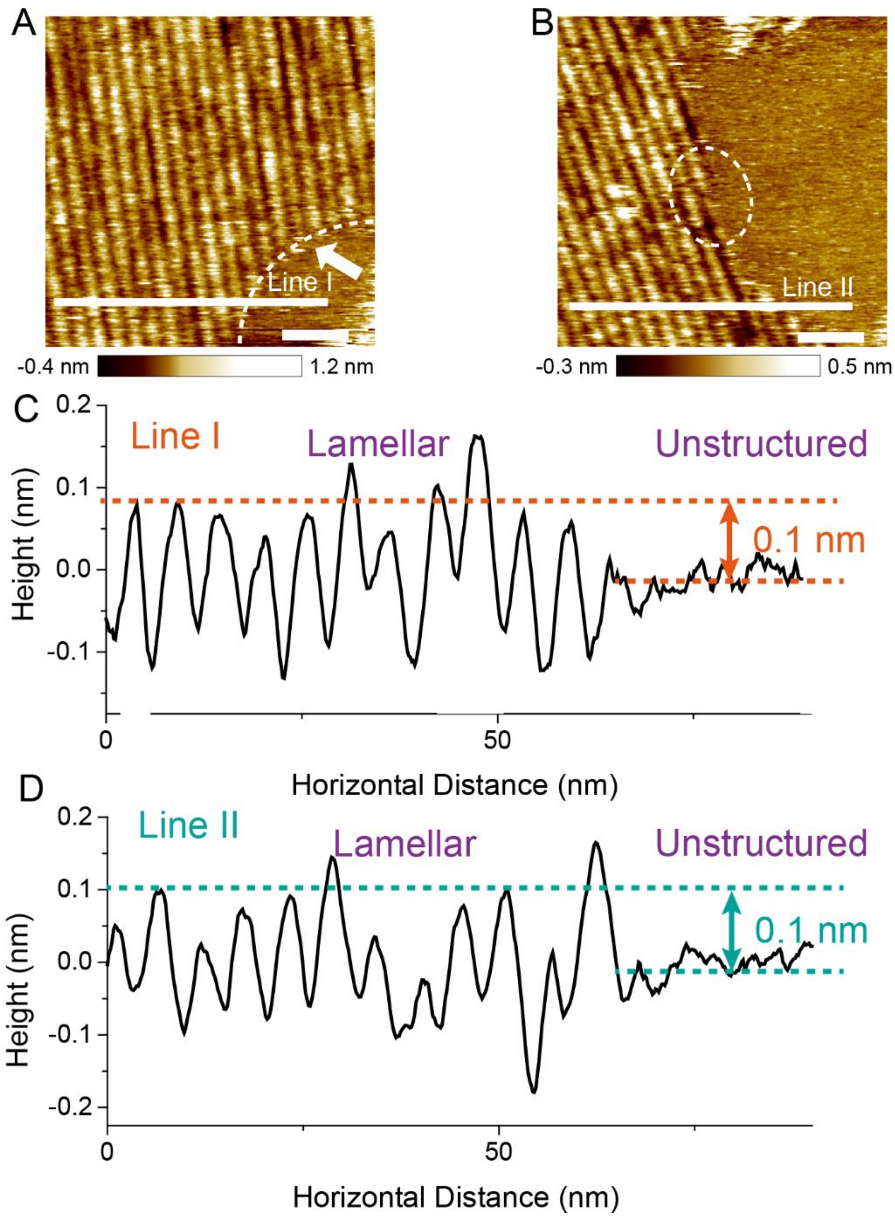

Fig. S20. The height information for lamellar structures. (**A** and **B**) Topography map two structures. (**C**) The height data of two structures (image A, line I). (**D**) The height data of two structures (image B, line II). Scale bar is 20 nm.

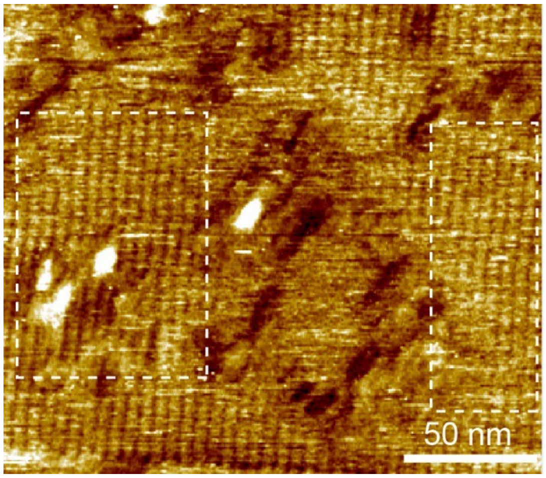

Fig. S21. AFM image of SF assembly. The white dash boxes mark unstructured regions that transformed into lamellar structures.

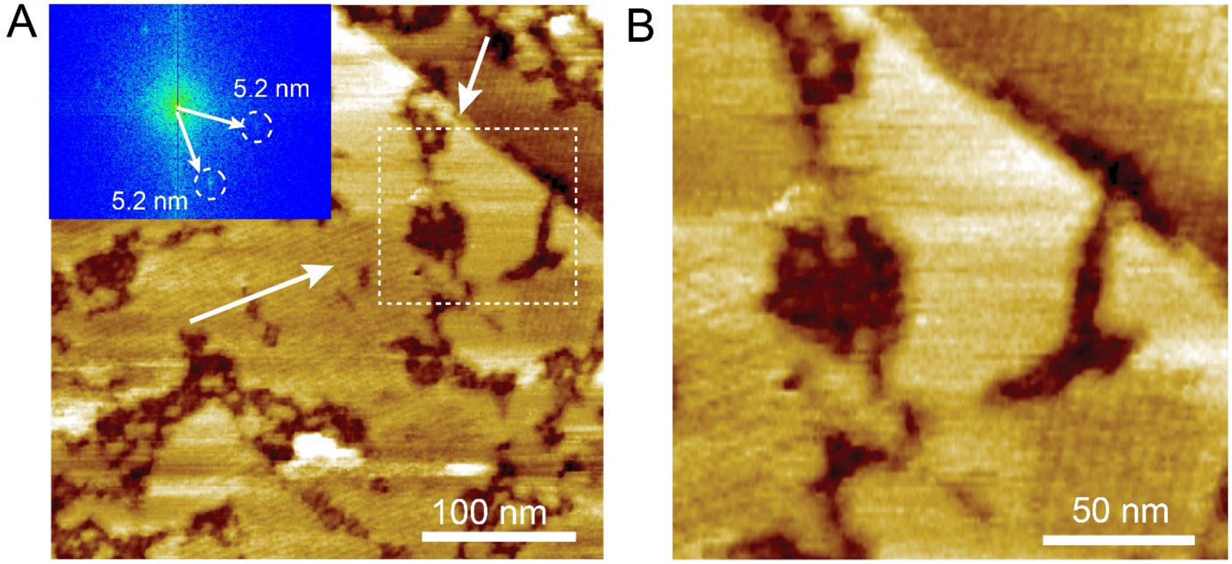

Fig. S22. Self-assembled film of SF formed at high concentration. The concentration of SF is 3 μg/mL. (**A**) The topography map of SF after 59 min of self-assembly, the inset is FFT image. (**B**) The corresponding topography map of white box in image (A).

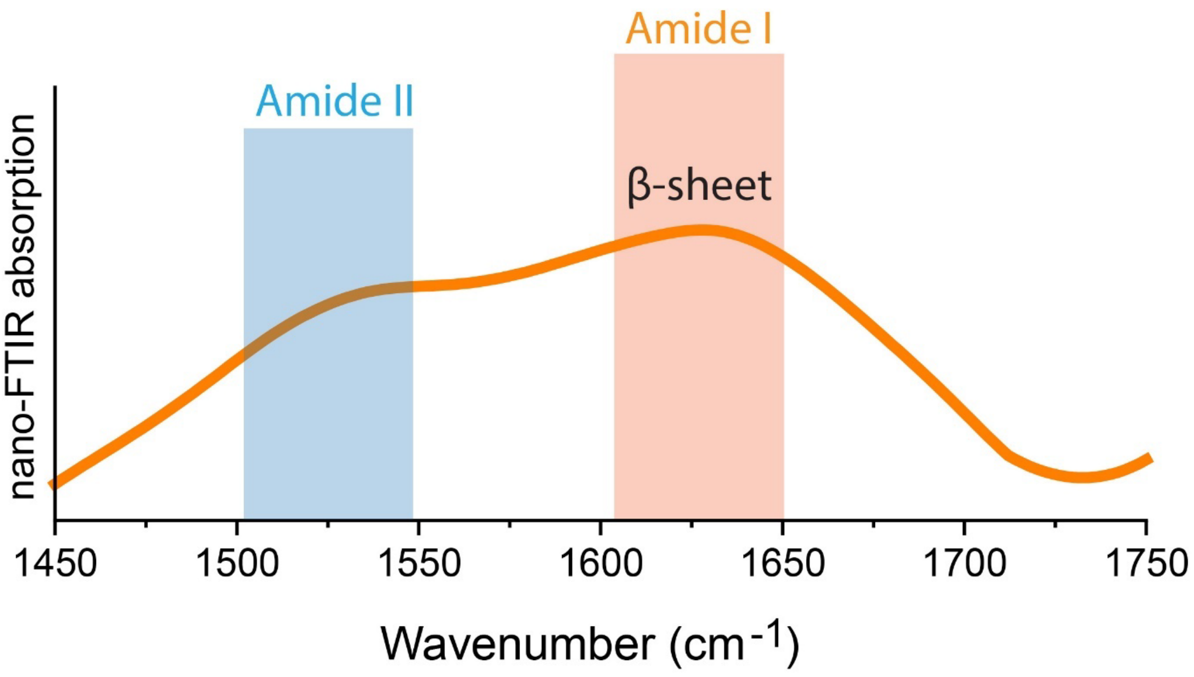

Fig. S23. Nano-FTIR spectra of an assembled SF structure in 3 μg/mL SF solution. The intensity of amide I signal higher than amide II during assembly in a concentration of 3 μg/mL SF solution, and the peak of amide I centered at 1630 cm^{-1}, representing the formation of more β-sheet structure, that is a thicker lamellar layer.

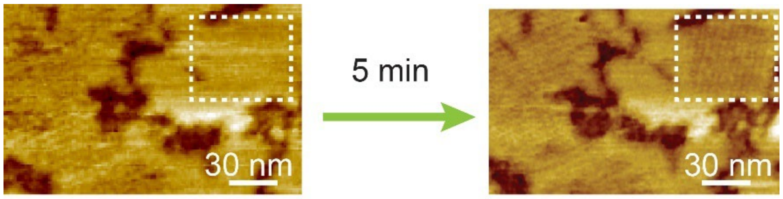

Fig. S24. Consecutive AFM images of assembly from 3 μg/mL SF solution showing the phase transition from unstructured to lamellar.

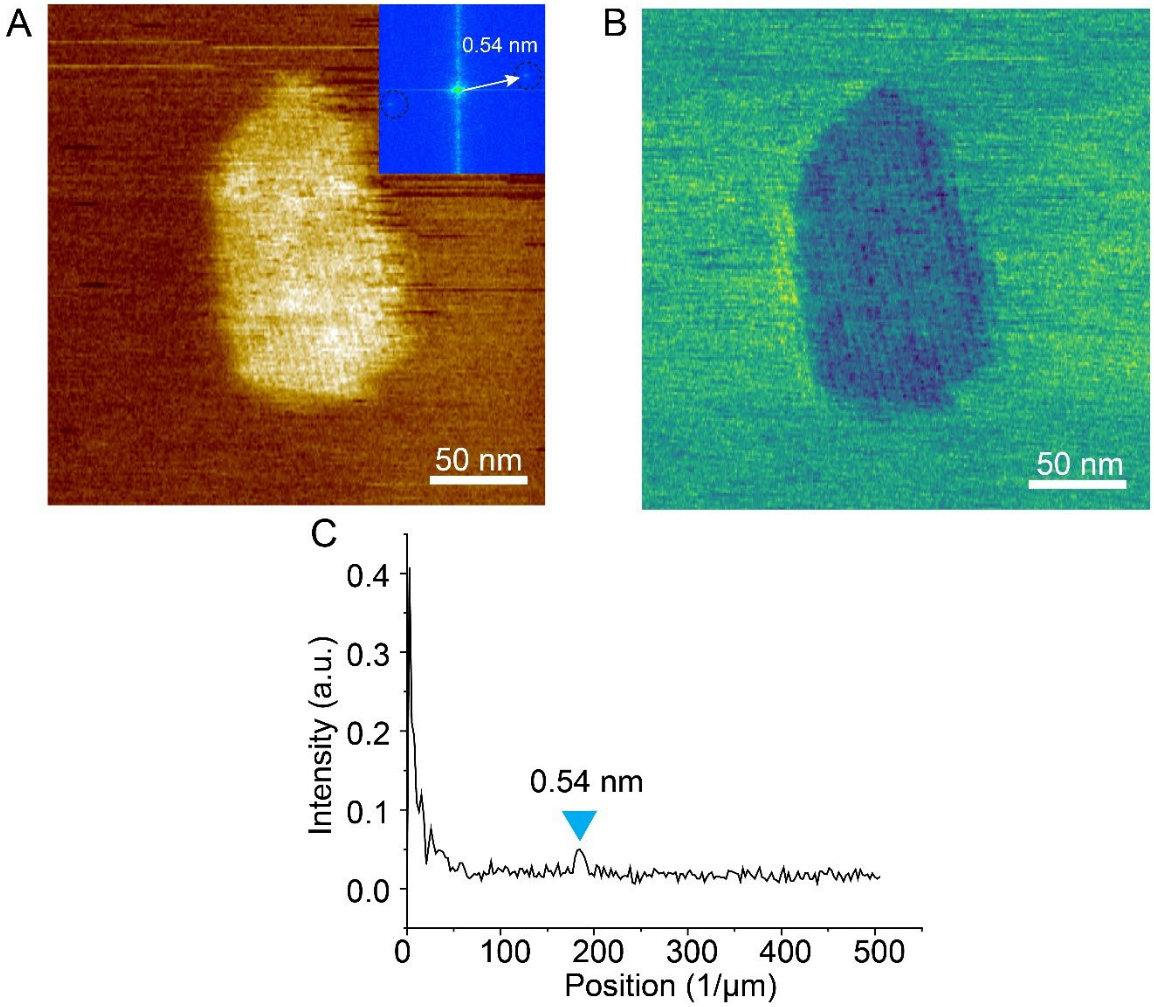

Fig. S25. The lamellar structure on MoS_2. (**A**) Morphology image. (**B**) Phase image. (**C**) 1D Fourier profile. The periodicity of lamellar on MoS_2 is same with HOPG, indicating a similar assemble process perform at the 2D surface. SF concentration: 0.05 μg/mL, incubation time: 30 min.

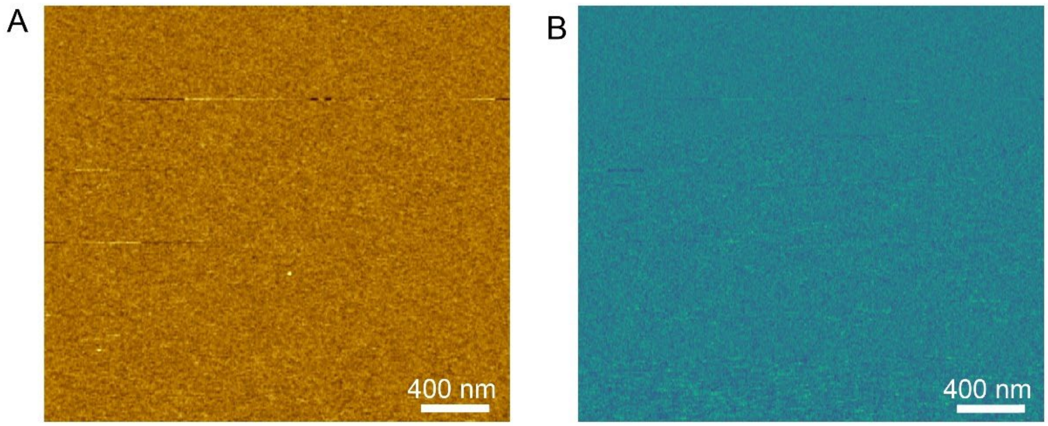

Fig. S26. 2D SF films do not form on Mica even after incubating 0.05 μg/mL SF for 60 min. (**A**) The height AFM image. (**B**) The phase AFM image.

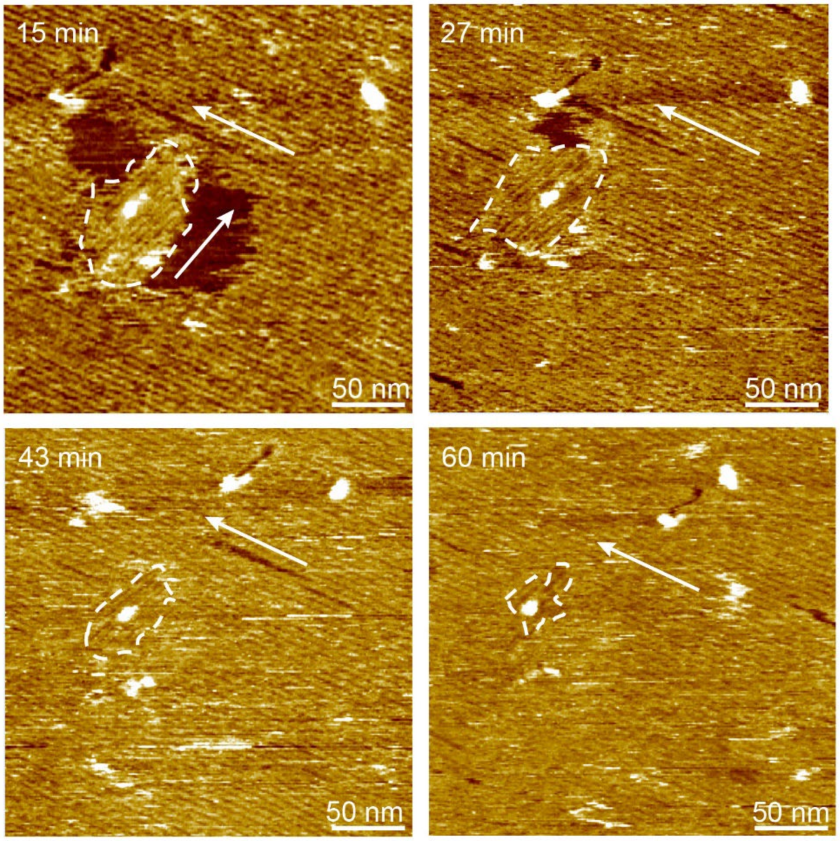

Fig. S27. Reorientation of lamellae. In situ AFM phase images showing lamellae twist their orientation as nearby lamellae grew. The growth direction in white dash box gradually replaced by another direction.

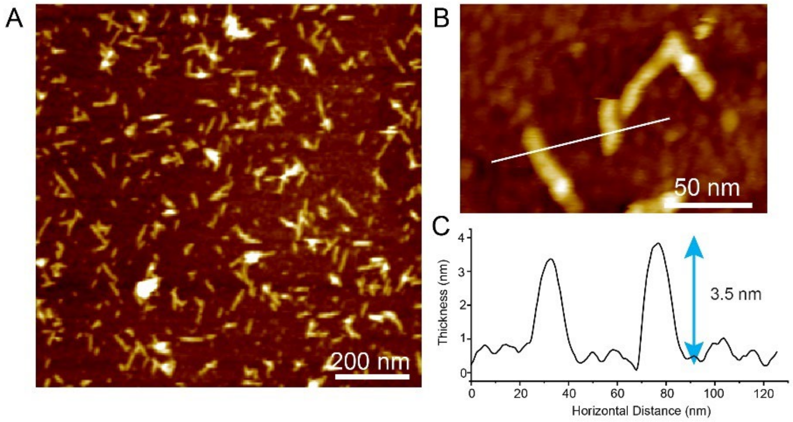

Fig. S28. Self-assembled SF nanofibrils formed at high concentration (30 μg/mL). (**A**) AFM images of SF nanofibril. (**B**) The high-resolution image of silk nanofibril. (**C**) The height of silk nanofibril (measured from the white line in image B).

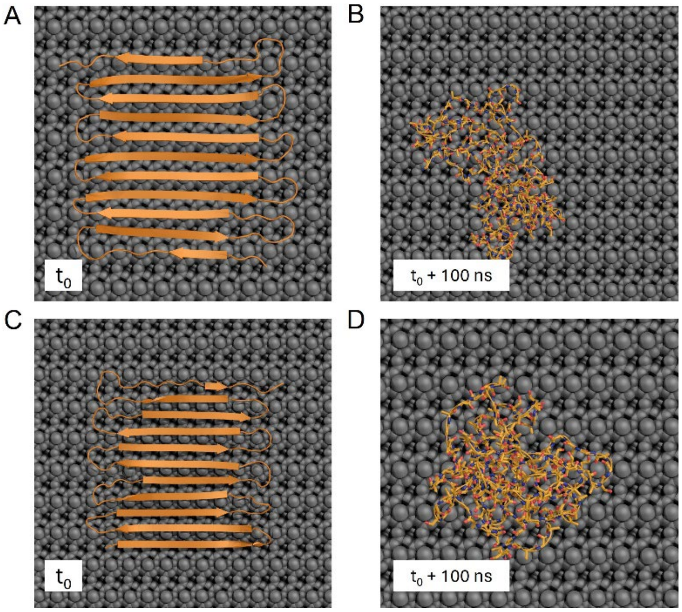

Fig. S29. Simulations predict that the SF secondary structure is not stable on mica. (**A** and **B**) Initial and final configurations of SF on Mica with sidechains facing the surface. (**C** and **D**) Initial and final configurations of SF on Mica with sidechains facing the bulk solvent. In all cases, SF quickly unbound and unfolded, in line with what would be expected from AFM results.

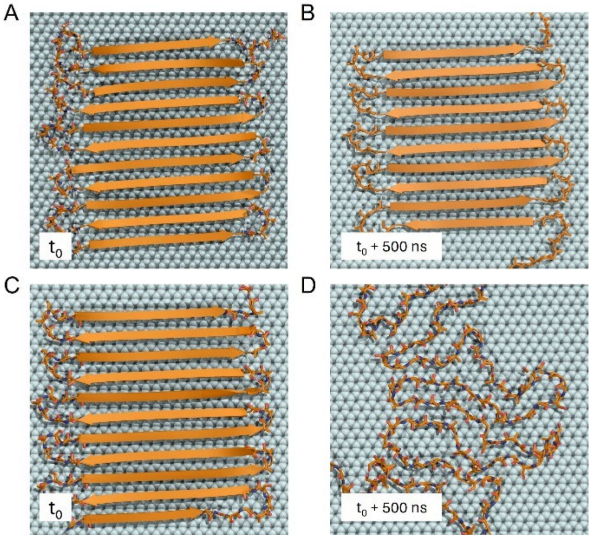

Fig. S30. Simulations of SF β-sheets binding to HOPG. SF β-sheet was placed ~1 nm above graphite and allowed to bind. The protein initially aligned along the armchair edge. (**A** and **B**) show SF with alanine sidechains facing bulk solution. (**C** and **D**) show SF with alanine sidechains facing the surface. Note that these results have previously been replicated when the protein was placed directly on the surface, with no interfacial waters (*20*).

Table S1. The height of a lamellae layer was determined by extracting the z-coordinate of the layer's non-turn residue backbone center of mass. For the monolayer lamellae, thickness was calculated relative to the z-coordinate position of carbon atoms in the first layer of graphene. For the bilayer lamellae, thickness was calculated by subtracting the height of the lower layer from that of the upper layer. Thickness was averaged over the last 90 ns of production simulation time for all systems.

System	Height (Å)
Face-to-face double layer	3.94 ± 0.077
Face-to-back double layer	3.26 ± 0.028

Table S2. The basic parameter of the simulations model.

System	Notes	Size (nm)	Simulation Time	Replicas
**Single protein alanine SCs up along armchair**	Protein secondary structure restrained during equilibration/binding	10.4x10.3x10.5	125 ps NVT, 50 ps NPT, 500 ns NVT	3
**Single protein alanine SCs up along zigzag**	Protein secondary structure restrained during equilibration/binding	10.4x10.3x10.6	125 ps NVT, 50 ps NPT, 100 ns NVT	3
**Single protein alanine SCs up along intermediate angle**	Protein secondary structure restrained during equilibration/binding	10.4x10.3x10.7	125 ps NVT, 50 ps NPT, 100 ns NVT	3
**Single protein alanine SCs down along armchair**	Protein secondary structure restrained during equilibration/binding	10.4x10.3x10.8	125 ps NVT, 50 ps NPT, 500 ns NVT	3
**2x2 bilayer upper layer alanine SCs up**	Inter-protein waters placed during solvation were deleted	11.3x10.7x6.7	125 ps NVT, 300 ps NPT, 100 ns NVT	3
**2x2 bilayer upper layer alanine SCs down**	Inter-protein waters placed during solvation were deleted	11.3x10.7x6.7	125 ps NVT, 300 ps NPT, 100 ns NVT	3
**Unfolded protein on lamallae**	System size decreased after binding	9.9x20.5x8.7, 8.4x10.7x6.5	125 ps NVT, 50 ps NPT, 1000 ns NVT	3
**Unfolded protein on hopg**	None	10.4x10.3x7.5	125 ps NVT, 50 ps NPT, 1000 ns NVT	3
**Single protein alanine SCs up on mica**	Protein secondary structure restrained during equilibration	10.3x9,9x9.8	125 ps NVT, 50 ps NPT, 100 ns NVT	3
**Single protein alanine SCs down on mica**	Protein secondary structure restrained during equilibration	10.3x9,9x9.8	125 ps NVT, 50 ps NPT, 100 ns NVT	3
